# A Survey on Heterogeneity Taxonomy, Security and Privacy Preservation in the Integration of IoT, Wireless Sensor Networks and Federated Learning

**DOI:** 10.3390/s24030968

**Published:** 2024-02-01

**Authors:** Tesfahunegn Minwuyelet Mengistu, Taewoon Kim, Jenn-Wei Lin

**Affiliations:** 1 Department of Information Convergence Engineering, Pusan National University, Busan 46241, Republic of Korea; tesfahunegn9@pusan.ac.kr; 2 Department of Computer Science and Information Engineering, Fu Jen Catholic University, New Taipei City 242062, Taiwan; jwlin@csie.fju.edu.tw

**Keywords:** Internet of Things, wireless sensor networks, federated learning, heterogeneity, machine learning, security and privacy, energy efficiency

## Abstract

Federated learning (FL) is a machine learning (ML) technique that enables collaborative model training without sharing raw data, making it ideal for Internet of Things (IoT) applications where data are distributed across devices and privacy is a concern. Wireless Sensor Networks (WSNs) play a crucial role in IoT systems by collecting data from the physical environment. This paper presents a comprehensive survey of the integration of FL, IoT, and WSNs. It covers FL basics, strategies, and types and discusses the integration of FL, IoT, and WSNs in various domains. The paper addresses challenges related to heterogeneity in FL and summarizes state-of-the-art research in this area. It also explores security and privacy considerations and performance evaluation methodologies. The paper outlines the latest achievements and potential research directions in FL, IoT, and WSNs and emphasizes the significance of the surveyed topics within the context of current technological advancements.

## 1. Introduction

The growing data generated by billions of connected IoT devices are causing challenges in traditional machine learning (ML) approaches. Centralised approaches, like those used by major tech companies such as Facebook and Google, can potentially compromise user privacy by sending personal data to a central data centre. However, FL can train models without sharing data but may face unique challenges in communication, computation, privacy, storage, power, and energy utilisation. The IoT paradigm is effective in managing these challenges, but it also raises issues including network attacks, data theft, and energy consumption. The rise in wireless communication technology has significantly increased the need for intelligent computing, leading to extensive research on network learning using artificial intelligence algorithms [1].

Recent advances in deep learning (DL) and ML in particular have brought about a radical shift in our relationship with modern digital devices. Even a few years ago, we would never have imagined that deep learning applications would lead to the creation of virtual assistants such as Alexa, Siri, and Google Assistant, or that self-driving cars would be a reality. But today, these innovations are a regular part of our existence. Massive training infrastructures and training data sets must be readily available for this to be successful in large part. However, as ML users and service providers become more aware of the privacy consequences of this data-hungry process, government regulators and service providers have developed several initiatives to safeguard citizens’ privacy. Aside from the privacy implications, with regard to data locality, data must be processed at the original location of collection and storage due to energy efficiency and climate change considerations. This is evolving into a key element of ML. FL is a rapidly developing field of ML that enables model training without transferring data from users’ devices. This approach has the potential to overcome these challenges. On the other hand, FL moves the computation to the clients’ devices so that multiple users can collaborate on training a single model, and it has long been thought that computation should be moved to a dispersed edge device [2].

The integration of billions of IoT and WSN devices has led to a significant increase in their usage. These devices, such as robots, drones, and smartphones, have limited computational and storage capacities but can communicate with distant entities through wide-area networks (WAN). As a result, the amount of data produced by these devices at the network edge is growing exponentially. However, due to bandwidth and privacy concerns, it is not practical to send all this data to the server. Many IoT and WSN applications require data prediction and classification, which necessitates training ML models using data from multiple devices. The challenge is how to use decentralised data from resource-constrained IoT devices to train ML models without transferring raw data between client devices. To address this challenge, FL has gained much attention, which is a method that allows learning to occur without transferring raw data. FL enables each device to gain a global perspective and predict events observed by other devices. However, implementing FL in the presence of heterogeneity poses difficulties. To overcome these difficulties, an FL framework is proposed that considers heterogeneous edge clients. This framework utilises a soft-training optimisation method that dynamically masks neurons based on model updates. Additionally, an aggregation scheme is suggested to expedite collaborative convergence and address issues with straggler clients. However, there is a lack of thorough analysis of FL problems and challenges in the context of heterogeneity and integration with WSNs and IoT. In order to implement FL on heterogeneous systems and integrate it with IoT and WSNs, it is crucial to analyse the fundamental problems, viable solutions, and future directions [3].

We believe that there is a gap in the literature regarding the architecture and systems of the IoT, WSNs, and FL that are already implemented and that motivate developers to create solutions that combine all three. This paper offers several notable contributions compared to previous surveys:

1. We conduct a comprehensive analysis of the integration of IoT, WSNs, and FL from various angles, such as system components, classification, and design.
2. We introduce a refined taxonomy to deal with heterogeneity in five different dimensions: statistical heterogeneity, device heterogeneity, architectural heterogeneity, model heterogeneity, and network and communication heterogeneity. This new taxonomy will help understand the current state-of-the-art in heterogeneous FL methods.
3. We discuss the heterogeneity issues that are essential for successful FL and thoroughly examine each case.
4. We review existing studies in different domains to provide a handy reference for researchers and developers.
5. We perform a complete analysis of security and privacy issues.
6. We suggest performance evaluation methods that use various metrics to assess system performance, such as latency, energy consumption, scalability, accuracy, and communication overhead.
7. We identify important research topics and challenges for future FL generations.

This paper is organised as follows. Besides the introduction, we sketch an overview of FL, which includes the basic knowledge, principles, and categories of FL based on strategies and types in Section 2. In Section 3, we present the integration of FL, IoT, and WSNs as well as discuss IoT, WSNs, and the opportunities and challenges of implementing FL in IoT and WSNs. Also, the realistic applications that integrate all three applications were discussed in various domains, including healthcare, smart cities, agriculture, industrial automation, and environmental monitoring. Additionally, we have addressed the integration of FL with sixth generation (6G) and digital twins. In Section 4, we discuss heterogeneity challenges in FL based on statistical heterogeneity, device heterogeneity, architectural heterogeneity, model heterogeneity, and network and communication heterogeneity, with a brief summary of the state-of-the-art research in heterogeneity. In Section 5, we point out security and privacy considerations in relation to threats and vulnerabilities and techniques for ensuring data confidentiality, integrity, and availability. We also draw conclusions on the use of current privacy-preserving measures and indirect information leaking in FL. In Section 6, we present the performance evaluation methodologies which help to assessing the effectiveness and efficiency of the integrated system involving the use of various metrics to measure system performance, including latency, energy consumption, scalability, accuracy, and communication overhead. In order to provide direction for future study, some frontier achievements are provided as the future research directions in Section 7. These discussions are centered around several promising FL, IoT, and WSN directions. Finally, Section 8 concludes the paper.

## 2. A Basic Knowledge of FL

In this section, we discussed how FL originated, how it works, and its classification based on different categories.

### 2.1. Background

The Google research team was the first to introduce FL. Their goal was to develop ML models that could be applied to the vast amounts of data that are available on mobile devices. FL was created to allow users to maintain control over their data and privacy. The device itself can be used to directly train the ML models in FL. The approach of applying the model to the data may be more appropriate in many cases, since the data may be sensitive and large in quantity. FL is the term coined to describe this decentralised method combined with collaborative learning [4].

Within the field of artificial intelligence, FL has grown quickly and gained prominence as a research area. Three important elements are responsible for this development. First and foremost, the widespread and effective use of ML technologies has facilitated the development of FL. Furthermore, the massive growth of big data has driven the demand for FL. Learning a global model while managing privacy concerns has become more difficult as large amounts of data are being stored on distinct devices by different organisations. FL is a growing alternative to traditional ML techniques, which are losing their effectiveness. Legislative constraints on data privacy have significantly benefited FL’s quick development. A major risk to user data privacy has emerged in the form of multiple data breaches in recent years. Several legislative laws have been created to address this, including the California Privacy Rights Act in the United States, the Singapore Personal Data Protection Act in Singapore, and the General Data Protection Regulation in the European Union. Particularly for privacy-preserving FL (PPFL), these regulations have significantly aided in the development of FL [5].

### 2.2. Brief Introduction to FL

**Figure 1:**
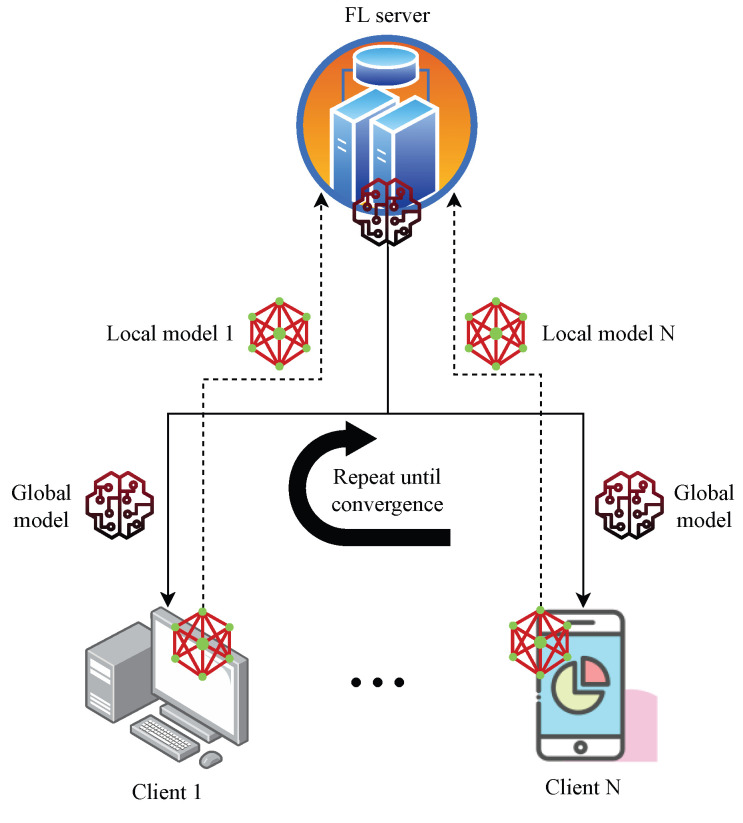
Federated learning concept and workflow.

A decentralised ML approach called FL allows a group of edge devices or nodes to collectively train a common model. With FL, the model can be trained locally on any device using local data instead of sending massive amounts of raw data to a central server for training, as shown in Figure 1. Only the model updates are sent back to the central server, ensuring privacy and reducing communication costs. FL is particularly useful in scenarios where data privacy is crucial or where there are limitations in terms of network bandwidth or latency [4,6,7,8].

In a FL system, the main participants are the clients that handle their local datasets, and the server that oversees the training process and updates the global model without accessing the client datasets. The model trained on each individual client is called the local model, while the model aggregated by the FL server is called the global model [5,9]. FL operates in the following manner:

Step 1. A central server initialises a baseline model weight $M_{W}^{0}$ and distributes it to the clients.
Step 2. Each client *x* trains the model $M_{x}^{k}$ in round *k* on its own local data and computes the model updates (such as the gradients or the weights). Obtaining the ideal local model parameters $W_{x}^{k^{*}}$ that decrease the loss function $lsf\left(M_{x}^{k}\right),\mathrm{where}\;M_{x}^{k^{*}}=arg\;min\;lsf\left(M_{x}^{k}\right)$, is the aim of the client x in round k.
Step 3. The clients send their model updates to the server.
Step 4. The server aggregates the model updates from the clients using some algorithm such as averaging $lsf\left(M_{W}^{k+1}\right)=\frac{1}{N}\sum_{x=1}^{N}lsf\left(W_{x}^{k}\right)$, where *N* is the number of clients and updates the global model.
Step 5. The server sends the updated global model $M_{W}^{k+1}$ back to the clients and repeats the process until convergence.

Examples of well-known FL algorithms are Federated Averaging (FedAvg), Federated Stochastic Gradient Descent (FedSGD), and FL with Dynamic Regularization (FedDyn). Additionally, a FL framework called FedCV was specifically created for computer vision applications, bridging the gap between research and practical application. It offers a unified library with a range of easy-to-use functionalities. FedCV has practical applications in the manufacturing, transportation, and healthcare sectors [10,11,12,13]. The detailed description and working principles of the FedAvg, FedSgD, and FedDyn are presented as follows:

The FedAvg algorithm is designed for distributed training with a large number of clients. Each client maintains its data locally to ensure privacy, while a central parameter server facilitates communication. The server distributes parameters to clients and collects model updates, requiring frequent communication between the server and clients as outlined in Algorithm 1 [5,9]. The algorithm in [5] works as follows: The input parameters are *R* (the maximum number of rounds), *m* (the number of clients selected in each round), $N_{\mathrm{epoch}\;}$ (the number of local epochs), η (the local learning rate), and $w_{G}^{0}$ (the initial global model parameters). *R* determines how many times the global model is updated by the server, *m* controls how many clients participate in the FL process, $N_{\mathrm{epoch}\;}$ specifies how many times each client iterates over its local dataset, η regulates how much the client model parameters are updated based on the local gradient, and $w_{G}^{0}$ is randomly initialised in a suitable range. Then it iterates for R rounds, where each round consists of the following steps. The server randomly selects a subset of *m* clients from the network and broadcasts the current global model parameters $w_{G}^{t}$ to them. Each selected client *i* performs the following operations in parallel: It divides its local dataset $D_{i}$ into batches and denotes the set of the batches by $B_{i}$. It performs $N_{\mathrm{epoch}\;}$ local epochs, where in each epoch it iterates over all the batches in $B_{i}$, and updates its model parameters *w* by taking a step in the direction of the negative gradient of the loss function L(w;b) evaluated on each batch *b*. It returns the updated model parameters $w_{i}^{t}$ and the size of its local dataset $N_{i}$ to the server. The server collects the model parameters and the dataset sizes from all the selected clients and computes the weighted average of the model parameters as the new global model parameters $w_{G}^{t+1}$, where the weights are proportional to the dataset sizes.The algorithm outputs the final global model parameters $w_{G}$ after R communication rounds.
**Algorithm 1:** FedAvg Algorithm [5]
1**Input:**2*R* : Maximum number of rounds.
3*m* : the number of clients selected in each round.
4$N_{\mathrm{epoch}\;}$ : the number of local epochs.
5η : the local learning rate.
6**Output:** Global model $w_{G}$
7**Processing:**8[Server-side]
9
Initialize $w_{G}^{0}$
10**for** *each round t from 1 to R***do** 
11    
    $S_{t}$ contains *m* clients randomly selected from the *n* clients
12    
    **for**
*each client $i\in S_{t}$
**in parallel***
**do**
13       $w_{i}^{t},N_{i}\leftarrow$**LocalTraining**$\left(i,w_{G}^{t}\right)$14

    **end**15


    $w_{G}^{t+1}=\frac{1}{\sum_{j=1}^{m}N_{j}}\sum_{i=1}^{m}N_{i}w_{i}^{t}$ [5]
16**end**17[Client-side]
18**LocalTraining**(i,w) :
19
Divide local dataset $D_{i}$ into batches; $B_{i}$ denotes the set of the batches.
20**for***each epoch j from 1 to $N_{epoch\;}$***do**21    
    **for**
*each batch $b\in B_{i}$*
**do**
22       w←w−η∇L(w;b)23        **end**24**end**25**return** the weights w and $N_{i}=\left|D_{i}\right|$


Federated Stochastic Gradient Descent (FedSGD) is a distributed optimisation technique that trains a ML model on multiple local datasets without sharing data samples. The main idea is to perform stochastic gradient descent (SGD) on each local node using its own dataset, and then periodically average the model parameters across all nodes as depicted in Algorithm 2 to obtain a global model. FedSGD can handle heterogeneous and non-IID data, as well as unreliable and resource-limited nodes [14]. The algorithm in [15] shows that running SGD in a federated setting can be viewed as adding a momentum-like term to the global aggregation process, and analyses the convergence rate of the algorithm by accounting for the effects of parameter staleness and communication resources. The algorithm works as follows. The input parameters are *H* (the number of local steps per communication round), η (the step size for SGD), and $w^{0}$ (the initial global model parameters). *H* determines how many times each client updates its local gradient estimation based on a randomly sampled data point from its own dataset; η controls how much the global model parameters are updated in each round based on the aggregated gradient from all clients; and $w^{0}$, which are randomly initialised in a suitable range. The algorithm iterates for *T* rounds, where each round consists of the following steps: The server randomly selects a $S_{t}$ of *N* clients and broadcasts the global parameter $w^{t}$ to them. Each selected client *k* performs the following operations in parallel: It initialises its local gradient estimation $g_{k}^{t,0}=0$; It performs *H* local steps, where in each step it samples a data point $\left(x_{i},y_{i}\right)$ uniformly at random from its own dataset $D_{k}$, where $D_{k}$ is the local dataset of client k; and updates its local gradient estimation $g_{k}^{t,s}$ by adding the gradient of the loss function $\nabla \ell \left(w^{t};x_{i},y_{i}\right)$ evaluated at the current global model parameters $w^{t}$ and it sets its final local gradient to the average of its local gradient estimations over the *H* steps $\left(g_{k}^{t}=g_{k}^{t,H}/H\right)$, and sends it back to the server. The server collects the local gradients from all the selected clients and assigns the previous local gradient $g_{j}^{t-1}$ to the unselected clients *j*. The server updates the estimation of the gradient $g^{t}=\sum_{k=1}^{K}\frac{n_{k}}{n}g_{k}^{t}$, where nk is the size of the local dataset of client *k* and *n* is the total number of data points. Also, the server updates the global model $w^{t+1}$ by taking a step in the direction of the negative aggregated gradient, scaled by the step size η : $w^{t+1}=w^{t}-\eta g^{t}$. The algorithm outputs the final global model parameters $W^{T}$ after *T* rounds.
**Algorithm 2:** Federated SGD Algorithm [15]
1**Input:**2H: number of local steps per communication round.
3η: step size for stochastic gradient descent.
4**Initialize:**  $w^{0}\in R^{d}$
5**for**  t=0,1,2,…,T−1 **do**
6    
  The server randomly selects a set $S_{t}$ of *N* clients and broadcasts the global parameter $w^{t}$ to them
7    
  **for**
*each client $k\in S_{t}$ in parallel*
**do**
8        
Initialize $g_{k}^{t,0}=0$
9        
  **for ***s=0 to H−1*
**do**
10           
Sample $i\in D_{k}$ uniformly at random, and update the local estimation of the gradient, $g_{k}^{t,s}$, as follows:
11           
    $g_{k}^{t,s+1}=g_{k}^{t,s}+\nabla \ell \left(w^{t};x_{i},y_{i}\right)$ [15]
12       
  **end**
13       
Set $g_{k}^{t}=g_{k}^{t,H}/H$ and send the parameter back to the server
14

  **end**15


The server collects all the updates of ${\left\{g_{i}^{t}\right\}}_{i\in S_{t}}$ and assigns $g_{j}^{t}=g_{j}^{t-1}$ for all $j\neq S_{t}$.
16


Then, the server updates both the estimation of gradient $g^{t}$ and parameter $w^{t+1}$ as follows:
17

    $g^{t}=\sum_{k=1}^{K}\frac{n_{k}}{n}g_{k}^{t}$,
18


    $w^{t+1}=w^{t}-\eta g^{t}$
19**end**20**Output:**  $w^{T}$



FL with Dynamic Regularization (FedDyn) is a novel FL method that trains a neural network model on multiple local datasets without sharing data samples. The main idea is to introduce a dynamic regulariser for each local node at each round so that the local and global solutions are aligned in the limit. FedDyn can handle heterogeneous and non-IID data, as well as unreliable and resource-limited nodes. The procedure of FedDyn is shown in Algorithm 3, and it works as follows: The input parameters are: *T* (total number of rounds), which determines how many times the global model is updated by the server; $\theta^{0}$ (the initial global model parameters), which are randomly initialised in a suitable range; α>0 (the regularisation parameter), which controls the trade-off between the local and global objectives; and $\nabla L_{k}\left(\theta_{k}^{0}\right)=0$ (the initial gradient at each client), which is set to zero. The parameters of the neural network, denoted as θ, are anticipated to be identical for both the client and the server. All client devices are denoted by k∈[m]. The server randomly samples a subset of *m* clients from the network and transmits the current global model parameters $\theta^{t-1}$ to them. Each selected client *k* performs the following operations in parallel: It minimises its local objective function $L_{k}\left(\theta \right)$ with a dynamic regulariser that depends on the previous gradient $\nabla L_{k}\left(\theta_{k}^{t-1}\right)$ and the previous global model parameters $\theta^{t-1}$, and obtains the updated model parameters $\theta_{k}^{t}$. It updates its gradient $\nabla L_{k}\left(\theta_{k}^{t}\right)$ by subtracting a term proportional to the difference between the updated model parameters $\theta_{k}^{t}$ and the previous global model parameters $\theta^{t-1}$. It transmits its updated model parameters $\theta_{k}^{t}$ to the server. For each unselected client *k*, the server sets its model parameters $\theta_{k}^{t}$ and gradient $\nabla L_{k}\left(\theta_{k}^{t}\right)$ to be the same as the previous round. The next two steps are to update the server’s auxiliary variable $h^{t}$ based on the difference between the aggregated selected client models and the previous global model, and then update the global model $\theta^{t}$ using a combination of the average of the selected client models and the auxiliary variable. The algorithm continues this process for *T* rounds, adapting the regularisation dynamically and aggregating information from selected clients in each round.The final global model parameters, $\theta^{t}$, will be output by the algorithm at completion of *T* rounds.

FedDyn combines elements of FL, dynamic regularisation, and model aggregation to collaboratively train a global model across the decentralised set of clients. The dynamic regularisation allows the model to adapt to local variations, and the aggregation process ensures collaborative learning while addressing staleness in the model updates. FedDyn addresses the challenge of achieving convergence in a neural network with identical structures across multiple clients and a central server. The proposed FedDyn in [16] method operates in rounds, where a subset of active clients receives the server’s current model and optimises a local empirical risk objective. This objective includes the local empirical loss and a dynamically updated penalised risk function based on both the local client model and the received server model. The local models are updated according to a minimisation process involving the penalised risk function. The key insight is that stationary points for client losses may not align with global losses due to data heterogeneity among clients. The method aims to reconcile the dual objectives of model convergence to a consensus and local updates optimising empirical losses, providing an intuitive justification for its design.

In conclusion, the FedAvg, FedSGD, and FeDyn algorithms are examples of FL techniques that enable multiple IoT devices to collaboratively train a shared model without sharing their raw data. FL is a distributed machine learning approach that allows each device to perform on-device training based on its local data and then communicate the model updates to a central server or other devices. In this way, FL can preserve the privacy of the data and reduce communication overhead and latency.

Some of the challenges of implementing FL on IoT devices are resource constraints, trust issues, and convergence problems. Different FL algorithms have different strategies to address these challenges. For example, FedAvg is a simple and widely used FL algorithm that runs a number of SGD steps on a small subset of devices and then averages the resulting model updates via a central server. FedSGD is a variant of FedAvg that reduces the communication frequency by sending the model updates after each SGD step. FeDyn is an FL algorithm that uses a trust mechanism and a reinforcement learning-based selection strategy to choose the devices that participate in the training process.
**Algorithm 3:** FedDyn Algorithm [16]
1**Input:**2T: Total number of rounds.
3**Initialize:**4$\theta^{0}:$ Initial global model parameters.
5α>0: Regularization parameter.
6$\nabla L_{k}\left(\theta_{k}^{0}\right)=0:$ Initial gradient at each client.
7**for **t=1,2,…T  **do**
8    
  Sample clients $P_{t}\subseteq \left[m\right]$ and transmit $\theta^{t-1}$ to each selected client,
9    **for** *each client $k\in P_{t}$ in parallel* **do**10       
Set $\theta_{k}^{t}=\underset{\theta }{\mathrm{argmin}}L_{k}\left(\theta \right)-\langle \nabla L_{k}\left(\theta_{k}^{t-1}\right),\theta \rangle +\frac{\alpha }{2}{∥\theta -\theta^{t-1}∥}^{2}$ [16]
11       
Set $\nabla L_{k}\left(\theta_{k}^{t}\right)=\nabla L_{k}\left(\theta_{k}^{t-1}\right)-\alpha \left(\theta_{k}^{t}-\theta^{t-1}\right)$
12       
Transmit client model $\theta_{k}^{t}$ to server
13

  **end**14


  **for**
*each client $k\notin P_{t}$, and in parallel*
**do**
15       
Set $\theta_{k}^{t}=\theta_{k}^{t-1},\nabla L_{k}\left(\theta_{k}^{t}\right)=\nabla L_{k}\left(\theta_{k}^{t-1}\right)$
16


  **end**
17


  Set $h^{t}=h^{t-1}-\alpha \frac{1}{m}\left(\sum_{k\in P_{t}}\theta_{k}^{t}-\theta^{t-1}\right)$
Set $\theta^{t}=\left(\frac{1}{\left|P_{t}\right|}\sum_{k\in P_{t}}\theta_{k}^{t}\right)-\frac{1}{\alpha }h^{t}$
18**end**

### 2.3. Basic Principles of FL

The primary goal FL is to leverage the collective intelligence of the edge devices without compromising user privacy or requiring data transfer to a central server [6,8,17]. The basic principles of FL can be summarised as follows.

#### 2.3.1. Model Aggregation

In FL, each edge device trains a local model using its own local dataset. These local models are then aggregated to create a global model that represents the knowledge learned from all participating devices. Model aggregation can be performed using various techniques such as averaging, weighted averaging, or more advanced methods such as secure multi-party computation (SMC) or homomorphic encryption [18,19].

#### 2.3.2. Privacy Preservation

One key advantage of FL is its ability to preserve user privacy by keeping the data on edge devices. Instead of sending raw data to a central server, only model updates or gradients are exchanged during the training process. This ensures that sensitive information remains on the device and reduces the risk of data breaches or unauthorised access [20,21,22].

#### 2.3.3. Communication Protocol

FL requires efficient communication protocols to exchange model updates between edge devices and the central server to minimise communication overhead and not to incur excessive delays in training. These protocols should be designed to minimise communication overhead while ensuring security and reliability. Commonly used communication protocols in FL include Secure Socket Layer (SSL), Transport Layer Security (TLS), and lightweight protocols like Message Queuing Telemetry Transport (MQTT) [23].

### 2.4. Categorisations of FL

There are different ways to categorise FL based on various criteria, such as the data distribution, the communication protocol, the learning objective, and the privacy level. In this paper, we presented categories of FL based on strategies, client settings, and data partition.

#### 2.4.1. Based on Strategies of FL

The three main strategies to perform FL are centralised aggregation-based FL, distributed aggregation-based FL, and hierarchical aggregation-based FL, as discussed below [13].

##### Centralised Aggregation-Based FL

**Figure 2:**
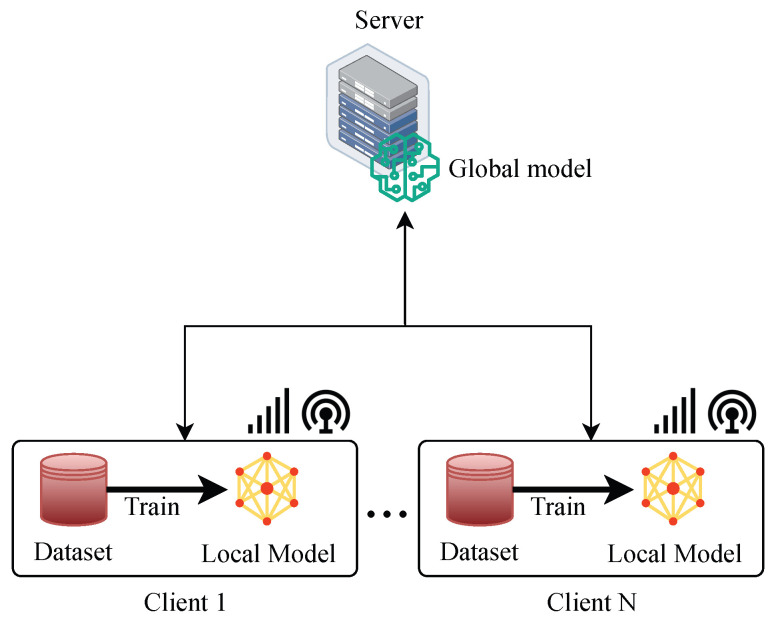
Centralised aggregation-based FL.

Centralised aggregation-based FL is a type of FL that uses a centralised server to aggregate the local models from multiple clients, as shown in Figure 2. The server sends the global model to all or a subset of clients in each round, and the clients update the model locally using stochastic gradient descent (SGD) or other optimisation methods. Then, the clients send their updated models back to the server, and the server aggregates them to obtain a new global model. This process is repeated until convergence or a predefined criterion is met. Centralised aggregation-based FL has some advantages over traditional centralised ML, such as reducing communication overhead, enhancing data privacy, and enabling distributed learning on heterogeneous devices [24,25,26,27]. However, it also faces some challenges, as follows:

1. The need for a reliable and secure server that can coordinate communication and aggregation among clients.
2. The vulnerability to malicious attacks or faulty clients that can compromise the global model or the aggregation process.
3. The difficulty of dealing with data heterogeneity and non-IIDness across clients can affect the convergence and accuracy of the global model.
4. The trade-off between communication efficiency and model performance which depends on the frequency and size of model updates.

##### Distributed Aggregation-Based FL

**Figure 3:**
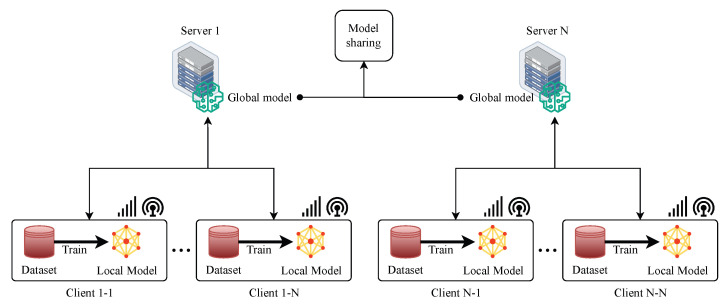
Distributed aggregation-based FL.

Distributed aggregation-based FL is a type of FL that does not rely on a centralised server to aggregate the local models from multiple clients as depicted in Figure 3. The fundamental concept is to divide the clients into groups and assign each group an aggregator that is responsible for collecting and aggregating the local models of the clients in that group. Then, the group aggregators communicate with each other in a peer-to-peer fashion to exchange and aggregate their models. The group aggregators then broadcast their aggregated models to the clients in their groups. This process is repeated until the global model converges. Distributed Aggregation-Based FL can achieve higher scalability, robustness, and privacy than centralised aggregation-based FL, as it eliminates the single point of failure and the bottleneck of communication [28,29,30,31]. However, it also introduces new challenges, such as:

1. There is a need for a reliable and secure distributed protocol that can coordinate communication and aggregation among clients.
2. Vulnerability to malicious attacks or faulty clients that can compromise the distributed protocol or the aggregation process.
3. The difficulty of dealing with data heterogeneity and non-IIDness across clients can affect the convergence and accuracy of the distributed model.
4. The trade-off between communication efficiency and model performance depends on the frequency and size of model updates.

##### Hierarchical Aggregation-Based FL

**Figure 4:**
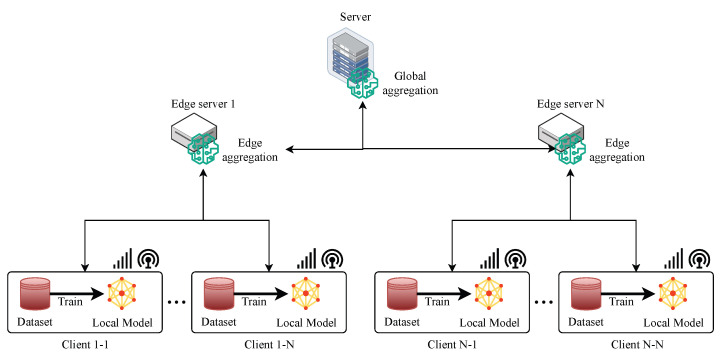
Hierarchical aggregation-based FL.

Hierarchical aggregation-based FL is a type of FL that leverages a hierarchical structure to aggregate the local models from multiple clients as shown in Figure 4. In hierarchical aggregation-based FL, there are multiple levels of aggregation, such as client–edge–global, where the clients communicate with the edge servers, and the edge servers communicate with the global server. The basic idea is to divide the clients into clusters and assign each cluster an aggregator that is responsible for collecting and aggregating the local models of the clients in that cluster. Then, the edge aggregators send their aggregated models to a global aggregator, which further aggregates them to obtain the global model. The global aggregator then broadcasts the global model to the edge aggregators, which in turn distribute it to the clients in their clusters. This process is repeated until the global model converges. Such an approach can reduce the communication overhead and latency, as well as improve the scalability and robustness of the system [28,32,33]. The basic steps of hierarchical aggregation-based FL are as follows:

1. The global server initialises a global model and sends it to a subset of edge servers that are selected randomly.
2. Each edge server updates the model locally by aggregating the models from a subset of clients that are connected to it.
3. Each edge server sends its updated model back to the global server.
4. The global server aggregates the received models, for example, by taking their weighted average, where the weights are proportional to the number of data points on each edge server.
5. The global server updates the global model with the aggregated model and repeats from step 1 until convergence.

#### 2.4.2. Based on Clients Setting

There are two different types of FL systems depending on the clients setting/scale of federation.

##### Cross-Silo FL Model

**Figure 5:**
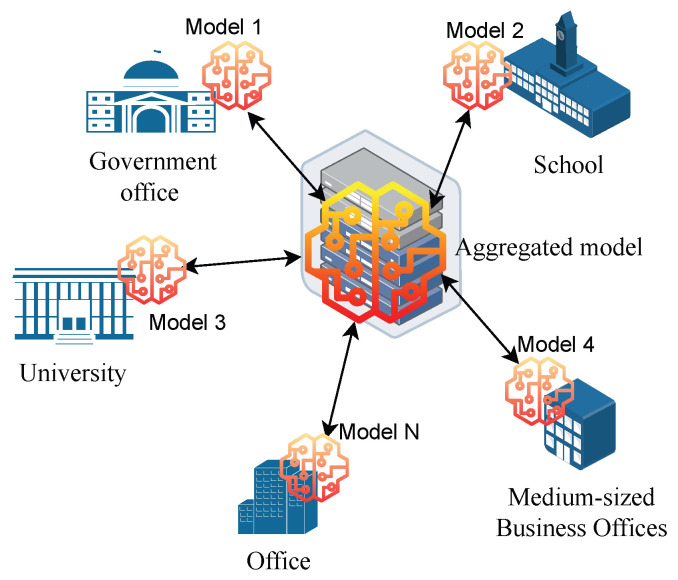
Cross-silo FL model.

When there are fewer participating devices that are available for every round, cross-silo FL is employed. Both vertical and horizontal FL formats are possible for the training set. Cross-silo is primarily utilised in situations involving organisations or data centres as shown in Figure 5.

Global Server: This server is responsible for coordinating the training process and aggregating the results from the different clients. Model 1 to Model N clients: These are the different organisations that are participating in the training process. Each client has its own data silo, which contains its own private data. Continuous Learning: The process of local model training, parameter updates, and global model aggregation continues iteratively until the desired level of accuracy is achieved for the overall model. In general, the working principle is as follows: The global server sends a copy of the current model to each client. Each client trains the model on its own data. Each client sends its model updates back to the server. The server aggregates the model updates from all of the clients. The server sends the aggregated model back to all of the clients. This process is repeated until the model converges.

Cross-silo FL has a number of advantages over traditional machine learning. (i) Data Privacy: Participating organisations retain control over their own data, never having to share it directly with other participants. (ii) Collaboration: Multiple organisations can pool their data and expertise to train a more accurate and generalisable model than any individual organisation could achieve alone. (iii) Reduced Costs: Organisations do not need to invest in the computational resources required to train the model from scratch, as the training is distributed across all participants.

However, there are also a number of challenges associated with cross-silo FL. (i) Communication Overhead: The constant communication between participants and the central coordinator can be computationally expensive and bandwidth-intensive. (ii) Data Heterogeneity: Data across different organisations might not be identically distributed, which can lead to challenges in model training and convergence. (iii) Incentivisation: Ensuring fair and equitable participation among organisations can be difficult, as some might contribute more data or resources than others.

In general, cross-silo FL is a promising approach that has the potential to revolutionise the way we train machine learning models. However, there are still a number of challenges that need to be addressed before it can be widely adopted. The cross-silo systems have low scalable federation. If stabilised, it is high; they also has a high-performance computation and storage capacity. Usually, the data distribution is non-IID [34,35].

##### Cross-Device FL

**Figure 6:**
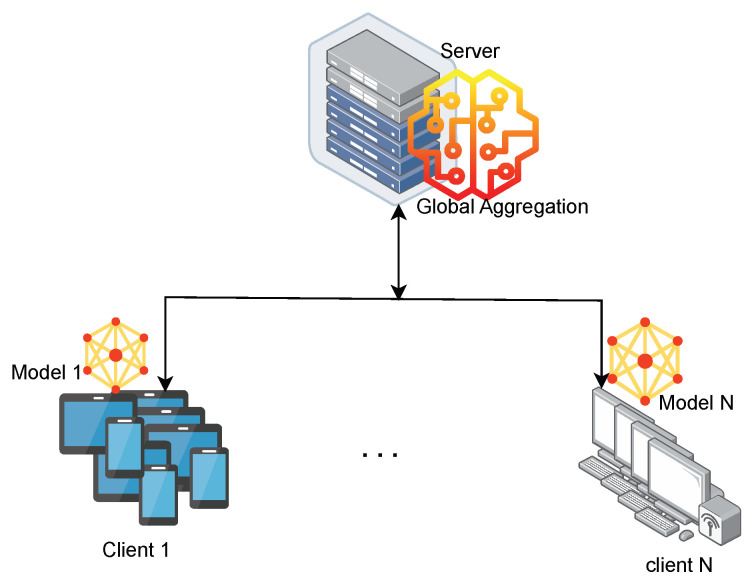
Cross-device FL model.

Cross-device FL is used in scenarios where a large number of devices participate, as illustrated in Figure 6. The design of incentives and client selection are two important strategies required to support this kind of FL. They are always able to be enabled and disabled. Typically, these are IoT and mobile devices.

The illustration depicts a central server communicating with multiple client devices (Client 1 to Client *N*), which are various IoT devices. The objective is to collaboratively train a machine learning model without directly sharing the data from each individual device. In the FL system, the central server acts as the coordinator, overseeing the entire cross-device FL process. It provides the initial global model to all client devices and aggregates updated local model parameters from clients to form a new, improved global model. Then, it sends the updated global model back to all client devices for the next training round. Regarding the client devices (IoT Gadgets), each device possesses its own local data and trains a local model (Model 1, ..., N) based on its data. A device sends updated local model parameters to the central server after each training round and then receives the newly aggregated global model from the server for the next training round. For the global model, the initial model was distributed to all devices for local training. It continuously improves with each round of aggregation, incorporating knowledge from all participating devices. Each local model (Model 1, ..., N) is trained on each device’s individual data, capturing unique insights and patterns. The updated parameters are sent to the central server for global model aggregation.

Key advantages of cross-device FL are as follows. (i) Data Privacy: Individual device data stays on the device, preserving privacy while enabling collaborative learning. (ii) Scalability: can involve a vast number of diverse IoT devices, leading to richer and more generalisable models. (iii) Resource Efficiency: The training burden is distributed among devices, reducing resource requirements for individual units.

The challenges of cross-device FL are as follows. (i) Communication Overhead: Frequent communication between devices and the server can strain bandwidth and battery life. (ii) Device Heterogeneity: Differences in device capabilities and data distributions can pose challenges for efficient aggregation. (iii) Incentivisation Mechanisms: Ensuring fair participation and contribution from all devices can be complex.

In conclusion, cross-device FL presents a promising approach for utilising the collective power of numerous IoT devices in machine learning tasks while addressing data privacy concerns and resource limitations. Based on stability, computation, and storage capacity, it is low, whereas based on scale, it is larger, and data distribution is usually IID [34,35]. As research and development in this area progresses, we can expect to see advancements in addressing the current challenges and unlocking the full potential of cross-device FL for various applications.

#### 2.4.3. Based on Data Partition

FL systems are generally classified as horizontal, vertical, or federated transfer learning systems, based on the distribution of data [5,12,34].

##### Horizontal FL

**Figure 7:**
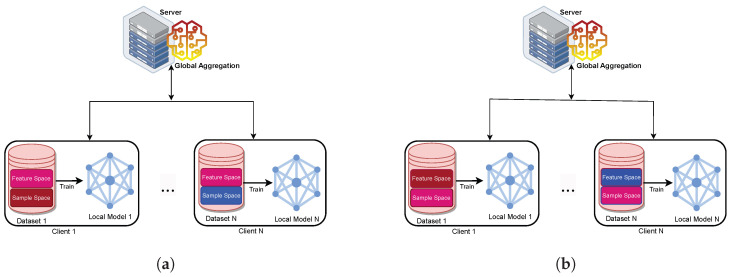
Horizontal and vertical federated learning. (**a**) Horizontal FL where sample spaces are the same but with different feature spaces among the devices; (**b**) Vertical FL where devices have different feature spaces but with the same sample spaces.

Horizontal FL is a type of FL that is suitable for scenarios where the data sets have the same feature space but different sample ID spaces, as shown in Figure 7a. This type of learning is applied in FL’s first application, the Google keyboard, where the participating mobile phones have distinct training data but share the same characteristics. The corresponding data and feature characteristics can be formally defined as follows: $X_{\mathrm{feature}\;}^{i}=X_{\mathrm{feature}\;}^{j}\;\mathrm{and}\;X_{ID}^{i}\neq X_{ID}^{j}\;\mathrm{for}\;\forall D_{i},D_{j},i\neq j$. Where, $X_{ID}^{i}$ is the set of unique identifiers of the samples in the ith party’s dataset $D_{i}$ and $X_{\mathrm{feature}\;}^{i}$is the set of features of the samples in the ith party’s dataset $D_{i}$. $X_{\mathrm{feature}\;}^{i}=X_{\mathrm{feature}\;}^{j}$ signifies that the feature spaces employed for training the ML model across different clients $D_{i}$ and $D_{j}$ are identical, ensuring consistency in the representation of data features. Concurrently, $X_{ID}^{i}\neq X_{ID}^{j}$ imposes the condition that the sample or data IDs from one client are distinct from those of another client, preserving the privacy of individual data. The entire equation is encapsulated within the scope of the universal quantifier $\forall D_{i},D_{j},i\neq j$ emphasising that these conditions universally apply to all pairs of distinct clients involved in the FL process. Therefore, Horizontal FL involves collaboratively training a model across multiple clients, where the features are shared, but the actual data samples are unique to each client, promoting both consistency and privacy in the learning process [36].

##### Vertical FL

As seen in Figure 7b, vertical FL is used when each device contains datasets with distinct feature spaces but the same sample ID spaces. For example, Vertical FL can be used to build a shared ML model when two organisations have data about the same population but different feature sets. In this regard, the characteristics of the dataset can be stated as follows: $X_{ID}^{i}=X_{\mathrm{ID}}^{j}\;\mathrm{and}\;X_{\mathrm{feature}\;}^{i}\neq X_{\mathrm{feature}\;}^{j}\;\mathrm{for}\;\forall D_{i},D_{j},i\neq j$. The $X_{ID}^{i}=X_{ID}^{j}$ asserts that the sample or data IDs from different clients $D_{i}$ and $D_{j}$ are equal, indicating a shared identity space among clients. Meanwhile, $X_{\mathrm{feature}\;}^{i}\neq X_{\mathrm{feature}\;}^{j}$ specifies that the feature spaces used for model training across clients are distinct, reflecting variations in the data characteristics. Therefore, this framework involves collaborative learning across clients with shared data identities but differing feature spaces, allowing for the integration of complementary information without exposing the raw data, thus preserving privacy and promoting collaborative model training [37].

##### Federated Transfer Learning

**Figure 8:**
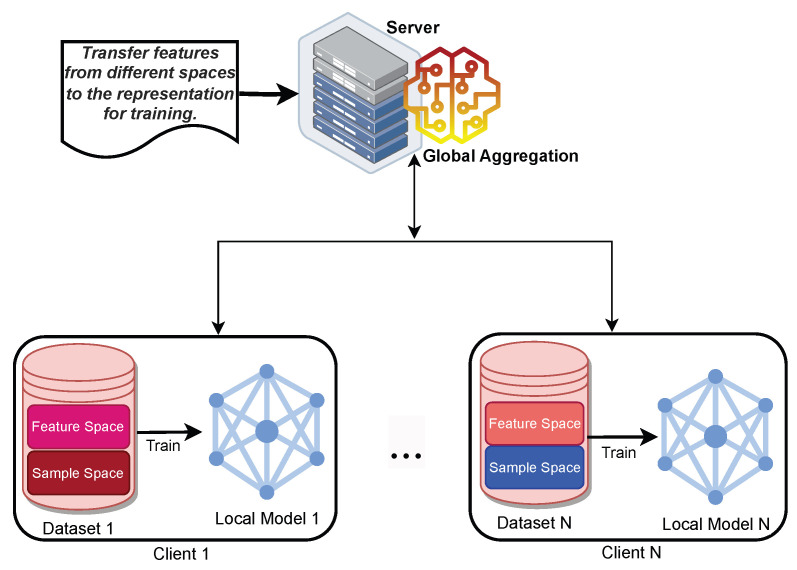
Federated Transferred Learning (FTL).

Federated transfer learning (FTL) is a combination of FL and transfer learning (TL) that allows knowledge to be shared and transferred among different parties without compromising user privacy. FL is a technique that enables multiple entities to collaboratively train a ML model without sharing their raw data. TL is a technique that enables an ML model to leverage the knowledge learned from one domain (the source) to improve its performance on another domain (the target). An ideal illustration of FTL would be to use a more comprehensive ML model that can learn from more data samples than each participating entity has access to. For example, suppose there are three parties, A, B, and C, that want to train a ML model for image classification. However, each party has a different set of images that do not have many overlapping features or labels. If they use FL alone, they may not be able to achieve good accuracy because of the data heterogeneity. If they use TL alone, they may not be able to preserve their data privacy because they have to share their data with the source domain. Therefore, they can use FTL to transfer the knowledge learned from each party to the others, while keeping their data local and secure, as seen in Figure 8. In this way, they can improve the ML model’s performance by leveraging the rich labels and features from each party. Formally speaking, the characteristics of federated transfer learning can be stated as follows: $X_{ID}^{i}\neq X_{ID}^{j}\;\mathrm{and}\;X_{\mathrm{feature}\;}^{i}\neq X_{\mathrm{feature}\;}^{j}\;\mathrm{for}\;\forall D_{i},D_{j},i\neq j$. The $X_{ID}^{i}\neq X_{ID}^{j}$ declares that the sample or data IDs from distinct clients $D_{i}$ and $D_{j}$ are not equal, signifying diverse data sources with unique identities. Simultaneously, $X_{\mathrm{feature}\;}^{i}\neq X_{\mathrm{feature}\;}^{j}$ specifies that the feature spaces utilised for model training across clients differ, capturing variations in the data characteristics. FTL enables collaborative knowledge transfer across clients with disparate data identities and feature spaces, facilitating the development of a more robust and generalised model that leverages collective intelligence from diverse sources while preserving data privacy [38].

## 3. Integration of FL with IoT and WSNs

In this section, we discuss the integration of IoT, WSNs, and FL. We have explored the opportunities and challenges involved in implementing FL in IoT and WSNs, as well as the application of the integrated systems. Additionally, we have addressed the integration of FL with 6G and digital twins.

### 3.1. IoT

**Figure 9:**
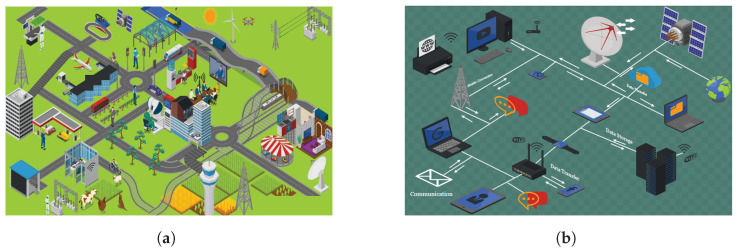
Illustration of IoT and WSN use cases. (**a**) Illustration of the IoT-based smart city with intelligent things; (**b**) Wireless technologies connecting diverse devices.

IoT is a concept that describes how different physical objects, such as cars, buildings, and other machinery, are connected to one another through network connectivity, software, and sensors. This interconnectedness allows for the seamless integration of the physical and digital worlds, leading to numerous potential applications and benefits across various domains, as depicted in Figure 9a. These connected devices collect and exchange data over the Internet, enabling them to interact with each other and with humans. IoT has gained significant attention in recent years due to its potential to revolutionise various industries and improve efficiency, productivity, and quality of life. The significance of IoT lies in its ability to connect various devices and systems, enabling seamless communication and data exchange. This connectivity opens up numerous possibilities for applications across various domains [39,40,41].

FL has been successfully applied in heterogeneous IoT environments. Here are some recent case studies and real-world applications:

- PervasiveFL is a framework that enables efficient and effective FL among heterogeneous IoT devices with different types of neural network models. It uses a lightweight model called Modellet on each device, which can learn from the local model and the global model using deep mutual learning and entropy-based decision gating. PervasiveFL can improve the inference accuracy of heterogeneous IoT devices with low communication overhead. It has been applied to image classification, face recognition, and natural language processing tasks [42].
- Model-heterogeneous FL is a method that allows clients to train models with varying complexities based on their hardware capabilities. It uses a novel aggregation scheme called model-aware federated averaging, which assigns different weights to different clients based on their model architectures and local data distributions. Model-heterogeneous FL can reduce the communication cost and improve the model’s performance in heterogeneous IoT environments. It has been applied to image classification and object detection tasks [43].
- ART4FL is an agent-based architectural approach for trustworthy FL in open, distributed, and heterogeneous IoT environments. It uses a multi-agent system to coordinate the FL process among different IoT devices and objects, which can dynamically join and leave the network. ART4FL can enhance the trustworthiness, security, and robustness of FL in heterogeneous IoT environments. It has been applied to smart cities and smart health scenarios [44].

These applications demonstrate the feasibility and effectiveness of FL in heterogeneous IoT environments, where devices can have different types of models, data, and resources. They also show the potential benefits of FL in terms of privacy preservation, data utilisation, and model generalisation. However, these applications also face some common challenges, such as how to deal with the non-IIDness, imbalance, and dynamicity of the data and devices, how to optimise the communication and computation trade-off, and how to ensure the security and reliability of the FL process. Some of the practical insights for future implementations should include:

- Consider the specific characteristics and requirements of the IoT applications, such as the type, size, and quality of the data and models, the availability and capability of the devices, and the communication and computation constraints.
- Explore the use of advanced techniques, such as compression, quantisation, sparsification, and encryption, to reduce the communication overhead and enhance the security and privacy of FL in heterogeneous IoT environments. Also, leverage the existing FL frameworks and platforms, such as TensorFlow Federated, PySyft, and FedML, to facilitate the development and deployment of FL in heterogeneous IoT environments.

### 3.2. WSNs

WSNs are an integral part of the IoT infrastructure. In order to monitor environmental or physical conditions, a vast number of inexpensive, small sensor nodes are placed in a particular area to form WSNs. These nodes communicate wirelessly, as shown in Figure 9b, with each other and with a central base station or gateway. WSNs enable real-time data collection from the environment and provide valuable insights for decision-making processes [45,46].

### 3.3. IoT and WSNs

In an IoT system, WSNs play a vital role in data collection and transmission. These networks consist of numerous sensor nodes that are distributed across a specific area or environment. Each sensor node is equipped with various sensors to measure physical parameters such as temperature, humidity, pressure, light intensity, and more. These nodes are typically battery-powered and have limited computational capabilities [47,48]. One major role of WSN in IoT is to collect sensed reading from the environment. Such data can then be used to train the intelligent service applications to be running on top of the IoT systems. The underlying physical sensors in WSN have a particular influence on FL and IoT, which is related to (i) which types of data can be collected by the sensors, and (ii) the reliability of the data collected by the sensors. The type of collectable data limits the coverage of the intelligent services to be offered, and the reliability of the sensed readings affects the accuracy of the FL model to be used as the basis of the intelligent service. The architecture of an IoT system involves multiple layers that facilitate the seamless integration of WSNs. At the bottom layer, we have the physical layer, which consists of the sensor nodes deployed in the environment. The upper layer is the network layer, responsible for managing communication between the sensor nodes and gateway devices. The gateway devices act as intermediaries between the sensor nodes and the higher-level layers of the IoT system [45,49].

The gateway devices in an IoT system are responsible for aggregating data from multiple sensor nodes and transmitting them to higher-level layers for further processing. They act as a bridge between the WSNs and other networks, such as local area networks (LANs) or wide area networks (WANs). The gateway devices can be connected to the internet or other communication networks to enable remote access and control of the IoT system [50]. To enable efficient communication within WSNs, various communication protocols are used. These protocols define how data is transmitted between sensor nodes and gateway devices. Some commonly used protocols in WSNs include Zigbee, Bluetooth Low Energy (BLE), Z-Wave, Wi-Fi, and Long Range Wide Area Network (LoRaWAN) [19,23,24,51].

Zigbee is a low-power wireless communication protocol created especially for WSNs. It offers dependable and secure communication between sensor nodes and gateway devices and runs on IEEE 802.15.4 standard [52]. Numerous industries, including healthcare, industrial monitoring, dand home automation, use Zigbee [53,54] Another well-liked communication protocol for WSNs is BLE. It is intended for short-range communication with minimal power consumption. Applications including wearable technology, asset tracking, and smart home systems frequently use BLE [55]. Z-Wave is a wireless communication protocol that operates in the sub-GHz frequency range. It is primarily used for home automation applications and provides reliable and secure communication between sensor nodes and gateway devices [56,57].

Wi-Fi, although not specifically designed for WSNs, can also be utilised in IoT systems. Wi-Fi provides high-speed data transmission over a relatively long range, making it suitable for applications that require real-time data processing and high bandwidth [58,59,60]. LoRaWAN is a low-power wide area network protocol that enables long-range communication between sensor nodes and gateway devices. LoRaWAN is well-suited for applications that require long-range connectivity, such as smart agriculture, smart cities, and asset tracking [61,62].

In addition to these wireless communication protocols, other protocols, such as MQTT and Constrained Application Protocol (CoAP), are used for efficient data transmission in IoT systems. These protocols are designed to minimise network overhead and power consumption while ensuring the reliable delivery of data [39,51,63]. IoT applications frequently employ MQTT, a lightweight publish–subscribe communications protocol. It makes it possible for sensor nodes and the gateway or cloud server to communicate effectively. MQTT uses a publish–subscribe model where sensor nodes publish data to specific topics and subscribers receive data from those topics [64,65]. Another low-power network protocol that is intended for devices with limitations is called CoAP. It makes it possible for devices with limited resources to communicate with the internet effectively. Sensor nodes act as clients in CoAP’s client–server model, transmitting and receiving data to and from servers [66].

In conclusion, the relationship between IoT and WSNs is symbiotic, with WSNs playing a crucial role in data collection and transmission within IoT systems. The architecture of IoT systems incorporates WSNs at the physical layer, with gateway devices acting as intermediaries between the sensor nodes and higher-level layers. Various communication protocols, such as Zigbee, BLE, Z-Wave, Wi-Fi, and LoRaWAN, are used to enable efficient communication within WSNs. Additionally, protocols like MQTT and CoAP are used for efficient data transmission in IoT systems.

### 3.4. FL in IoT

FL has gained significant attention in IoT environments due to its potential to address privacy concerns and scalability issues. In this section, we will explore the basic principles of FL, including model aggregation, privacy preservation, and communication protocols. Additionally, we will discuss the challenges and opportunities of implementing FL in IoT systems [19,51,67]. The general FL process includes the following key steps: system initialisation and device selection, where the aggregator chooses an IoT task such as human activity recognition and sets up learning parameters, e.g., learning rates and communication rounds; distributed local training and updates, where after the training configuration, the server initialises a new model and transmits it to the IoT clients to start the distributed training; finally, model aggregation and download, where after collecting all model updates from local clients, the server aggregates them and calculates a new version of the global model. The FL process is iterated until the global loss function converges or a desired accuracy is achieved [12].

#### 3.4.1. Opportunities of Implementing FL in IoT Systems

Despite the challenges to be introduced shortly, implementing FL in IoT systems presents several opportunities, such as:

**Privacy Preservation**: FL enables the training of models on sensitive data without compromising user privacy. This is particularly important in healthcare applications where personal health data needs to be protected [67,68]. To be specific, FL clients do not need to upload their data to the central server, and thus the vulnerabilities to security threats can be minimised, since the model updates are ephemeral and anonymous.

**Reduced Communication Overhead**: By keeping the data on edge devices and exchanging only model updates, FL reduces the amount of data transferred over the network. This leads to lower communication overhead and reduced latency, making it suitable for real-time applications in IoT environments [8]. Considering the general cases where the model size is much smaller than the entire dataset size, FL can save network bandwidth by exchanging the model updates only. However, if the model is complex and there are a large number of IoT devices participating in the same FL system and/or a large number of communication rounds are required for convergence, the FL system can also consume the network bandwidth significantly. Recent approaches, including local updating, compression schemes, and decentralised training, can effectively reduce communication overheads [69].

**Scalability**: FL allows for distributed training on a large number of edge devices simultaneously. This scalability makes it well-suited for IoT systems with a massive number of connected devices, such as smart cities or industrial IoT deployments [17]. For example, Bonawitz et al. [70] proposed a scalable TensorFlow-based production system for FL where FL clients are mobile devices. Also, Lee et al. [71] proposed a scalable FL system leveraging layer-wise adaptive model aggregation.

#### 3.4.2. Challenges of Implementing FL in IoT Systems

FL shows potential for training ML models in IoT environments, but there are several challenges that must be addressed. These challenges include limited resources, heterogeneity, and data imbalance [4,11,72]:

**Limited Resources**: IoT devices often have limited computational power, memory, and energy resources. Training complex ML models on these resource-constrained devices can be challenging. Optimising model architectures and developing lightweight algorithms are essential to overcome these limitations [73].

**Heterogeneity**: IoT systems are made up of a variety of devices with various operating systems, hardware configurations, and communication protocols. For FL to be implemented successfully, it is essential that these devices be compatible and are able to work together. Standardisation efforts such as the Open Connectivity Foundation (OCF) and the Thread Group aim to address these challenges [74,75,76,77].

**Data Imbalance**: In FL, the distribution of data across edge devices may not be uniform, leading to data imbalance issues. Some devices may have more representative or diverse datasets than others, which may have an impact on the global model’s performance. Techniques such as weighted aggregation or adaptive sampling can be employed to mitigate this problem [78].

### 3.5. FL in WSNs

Integrating WSNs with FL techniques can offer several advantages in terms of enhanced data analysis and decision-making in IoT systems. This integration allows for the efficient utilisation of resources, improved scalability, reduced communication overhead, and increased privacy and security. As expected, WSNs play an important role and have a significant influence on FL. In general, training a FL model requires multiple communication rounds among FL clients and the central server. If FL clients are heterogeneous with respect to the available set of sensors, the conventional FL approach cannot be applied. In addition, if FL clients are equipped with different types of communication modules or protocols, allowing all FL clients to participate in the same communication round can be challenging. In this section, we will discuss different approaches for combining WSNs and FL, including edge computing, data fusion, and resource allocation [11,50].

Edge computing is a paradigm that brings computation and storage capabilities closer to the edge of the network, where the data are generated. By deploying FL algorithms on edge devices within WSNs, it becomes possible to perform distributed learning tasks locally without transmitting raw sensor data to a central server. This reduces the communication overhead and improves response time, making it suitable for real-time applications. Edge computing also enables privacy-preserving FL by keeping sensitive data within the local network [18,19,79].

Data fusion is another approach for integrating WSNs with FL techniques. Data fusion involves combining information from multiple sensors to obtain a more accurate and reliable representation of the physical environment. By applying FL algorithms to fused sensor data, it becomes possible to train models that capture the collective intelligence of the sensor network. This can lead to improved accuracy in data analysis tasks, such as anomaly detection, classification, and prediction [80].

Resource allocation is an important aspect when integrating WSNs with FL techniques. Since WSNs typically operate under resource-constrained environments with limited energy, memory, and processing capabilities, efficient resource allocation becomes crucial. FL algorithms can be designed to adaptively allocate resources among sensor nodes based on their capabilities and the importance of the data they collect. This ensures that the most relevant and valuable data is used for training the models, while minimising resource consumption [81].

To summarise, integrating WSNs with FL techniques offers several benefits for data analysis and decision-making in IoT systems. Edge computing enables local and privacy-preserving FL, reducing communication overhead and improving response time. Data fusion allows for combining information from multiple sensors to obtain more accurate representations of the physical environment. Resource allocation ensures efficient utilisation of resources in resource-constrained WSNs.

#### 3.5.1. Opportunities of Implementing FL in WSNs

FL ensures data security and privacy by allowing learning tasks to be completed without requiring the sharing of raw sensor data. It also avoids sending a lot of data to a central server, which lowers communication overhead and energy consumption in WSNs. Furthermore, it leverages rich and diverse data from multiple WSNs to improve the robustness and accuracy of ML models. Moreover, by allowing WSNs to cooperate and share knowledge, it advances the creation of ubiquitous artificial intelligence in 6G communications [82,83,84].

#### 3.5.2. Challenges of Implementing FL in WSNs

Due to noise, interference, and bandwidth constraints in wireless channels, FL necessitates effective and dependable communication between WSNs, which can be difficult. Furthermore, due to the heterogeneous and asynchronous nature of WSNs, FL may encounter problems with model staleness and convergence. Different data distributions, computing power, and update rates may exist in these networks. Ferocious or compromised WSNs have the ability to introduce false data, alter model parameters, or deduce sensitive information from updates to the model, which raises security and privacy concerns in FL. In addition, the multitude and variety of WSNs, each with potentially different hardware, software, and communication protocols, can pose challenges for scalability and compatibility [69,85,86].

### 3.6. FL in 6G

FL is a crucial technology for 6G wireless networks, which aim to achieve widespread artificial intelligence (AI) in large-scale and diverse networks. The focus on FL in the context of 6G is justified for several reasons. Firstly, FL can improve the performance and efficiency of 6G networks by utilising distributed data and computation resources at the network edge. Secondly, FL can address the privacy and security concerns of 6G networks by avoiding the centralised collection and processing of sensitive data. Lastly, FL can enable the development and deployment of AI solutions for 6G networks by facilitating collaborative learning and inference among multiple devices [82,87]. FL can support various AI applications in 6G, including intelligent physical layer, intelligent edge computing, zero-touch network management, and intelligent resource management. Additionally, FL can also enable 6G use cases such as smart grid 2.0, Industry 5.0, and connected and autonomous systems [88].

### 3.7. FL in Digital Twins

A digital twin (DT) is a digital representation of a physical device or system, used for simulation, optimisation, and decision making. By integrating DTs with FL, a new architecture for IoT can be created. This allows DTs to capture the characteristics of IoT devices and assist FL in constructing a shared model. This integration offers several benefits, including reducing communication and computation overheads, adapting FL frequency and parameters based on the dynamic IoT environment, clustering IoT devices for asynchronous FL, and supporting industrial IoT use cases. FL and DT are complementary technologies that enhance the functionality and performance of IoT devices in various domains and scenarios [89,90,91,92,93].

### 3.8. Applications of Integrated IoT, WSNs, and FL

IoT, WSNs, and FL are interconnected technologies that have gained significant attention in recent years with applications in healthcare, smart cities, agriculture, industrial automation, and environmental monitoring. This section provides a comprehensive overview of their applications in these domains, discussing benefits and challenges [6,40,47,94].

**Healthcare**: The ability to monitor patients remotely via IoT, WSNs, and FL has revolutionised personalised medicine and efficient healthcare delivery. Wearables and medical sensors are examples of IoT devices that are used in remote patient monitoring to collect real-time health data from patients and transmit them to healthcare providers for analysis. This makes it possible to identify health problems early and take appropriate action. WSNs play a crucial role in healthcare by providing connectivity between medical devices and enabling seamless data transmission. FL techniques can be applied to analyse medical data collected from multiple sources while preserving data privacy. Yet, before these technologies are widely used in healthcare, issues including data security, interoperability, and regulatory compliance must be resolved [40,63,67].

**Smart Cities**: IoT, WSNs, and FL have immense potential for transforming cities into smart and sustainable environments. In smart cities, IoT devices are deployed to monitor various aspects, such as traffic flow, air quality, waste management, energy consumption, and public safety. WSNs play a critical role in collecting data from sensors deployed throughout the city and transmitting it to a central control system for analysis. FL techniques can be employed to analyse this vast amount of data collected from different sources while ensuring privacy and scalability. The integration of these technologies can lead to improved urban planning, resource optimisation, and enhanced quality of life. However, challenges related to data privacy, network scalability, and infrastructure deployment need to be addressed for successful implementation [18,23,40,41,95].

**Agriculture**: IoT, WSNs, and FL have the potential to revolutionise agriculture by enabling precision farming, crop monitoring, and livestock management. IoT devices such as soil sensors, weather stations, and drones can collect real-time data on soil moisture, temperature, humidity, and crop health. WSNs provide connectivity between these devices and enable seamless data transmission. FL techniques can be applied to analyse this data and provide insights for optimising crop yield, reducing resource consumption, and improving overall farm management. However, challenges such as limited network coverage in rural areas, power constraints for IoT devices, and data interoperability need to be addressed for widespread adoption in agriculture [96,97].

**Industrial Automation**: IoT, WSNs, and FL have transformed industrial automation by enabling real-time monitoring, predictive maintenance, and process optimisation. In industrial settings, IoT devices are deployed to collect data from various sensors and machines. WSNs provide connectivity between these devices and transmit the collected data to a central control system. FL techniques can be employed to analyse this data and provide insights for optimising production processes, reducing downtime, and improving overall efficiency. However, challenges such as network reliability, cybersecurity threats, and integration with legacy systems need to be addressed for successful implementation in industrial automation [98,99].

**Environmental Monitoring**: IoT, WSNs, and FL play a crucial role in environmental monitoring by enabling real-time data collection and analysis for better understanding of natural resources and ecosystems. In environmental monitoring applications, IoT devices such as weather stations, water quality sensors, and wildlife trackers collect data on various parameters such as temperature, humidity, pollution levels, and animal behavior. WSNs provide connectivity between these devices and transmit the collected data to a central control system for analysis. FL techniques can be applied to analyse this vast amount of data collected from different sources while ensuring privacy and scalability. This enables better decision-making for environmental conservation efforts. However, challenges related to power constraints for IoT devices in remote areas, data accuracy, and data integration need to be addressed for successful implementation in environmental monitoring [41,100,101].

In conclusion, IoT, WSNs, and FL have a wide range of applications in domains such as healthcare, smart cities, agriculture, industrial automation, and environmental monitoring. These technologies offer numerous benefits, such as real-time data collection, predictive analytics, and improved decision-making. However, challenges related to data security, interoperability, network scalability, and infrastructure deployment need to be addressed for successful implementation. The integration of these technologies has the potential to transform various industries and improve the quality of life for individuals worldwide.

### 3.9. A Summary of State-of-the-Art Research in FL

In this section we present the state-of-the-art research articles in FL under three categories according to the key concept discussed or covered therein. The Table 1 shows the summary of the related articles, and in this paper we classify these works into heterogeneity, security/privacy and other systems assisting FL such as IoT and WSN. Depending on the depth of the discussion regarding the aforementioned concept in each paper, the following three symbols, ✓, △, and ✗, are used to indicate comprehensive, partial, and little/no discussions.

## 4. Heterogeneity Challenge in FL

**Figure 10:**
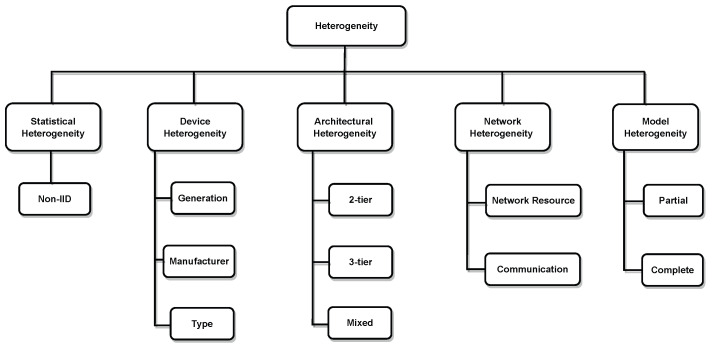
Proposed taxonomy of heterogeneity classifications in FL.

Heterogeneity in FL refers to the differences and diversity among participants or devices that collaborate to train a shared model without sharing their local data. FL faces the challenge of training on diverse data sets, devices, and networks that are beyond the control of the centralised FL server. This heterogeneity can cause the model to diverge, making the learning process ineffective. The heterogeneity in FL can stem from factors such as imbalanced data distribution, different hardware and network characteristics of client devices, unstable network connectivity, and limited device resources. In this paper, we present a taxonomy of the heterogeneity in FL by classifying it into five types, these being statistical, device, architectural, network and communication, and model heterogeneity, as shown in Figure 10.

### 4.1. Statistical Heterogeneity

Statistical heterogeneity in FL is a challenge that occurs when the data distributions of different clients are not identical and independent (non-IID). This can lead to bias in the global model or hinder convergence. As a result, it is critical for FL approaches to address statistical heterogeneity and create robust and efficient methods for aggregating local models or gradients from several clients [113,114]. Form distribution perspective statistical heterogeneity classified into label distribution skew, label preference skew, feature distribution skew, feature condition skew, label noise skew, sample noise skew and quantity skew. In FL, the data heterogeneity between two clients, *i* and *j*, can be measured by comparing their respective local data distributions, ci and cj. When conducting a supervised task in FL, a client is chosen randomly, and its local data distribution, ci(x,y), is used to extract feature-label pairs from (x,y) [68,72,115,116].

**Label distribution skew**: This implies that while the ci(x|y) scenario is the same, the label ci(y) distribution of various clients varies. For instance, in the distribution of client *i* on the MNIST dataset, 90% of the digits are 7 and 10% are other digits. In total, 95% of the values in the client *j* distribution are 7, and 5% of the numbers are other numbers. That is, there are differences in the distribution of ci(y). However, the comparable characteristic *x* has approximately the same likelihood of being 7 if *y* = 7. In other words, the distributions of ci(x|y) are identical.

**Label preference skew**: This suggests that the label distribution may vary for distinct clients, i.e., ci(y|x)≠cj(y|x), even in cases where the feature distribution is uniform across clients, i.e., ci(x) = cj(x). Label preference skew, or different labels for the same features, can result from horizontal overlap across local training datasets belonging to distinct clients. That is, due to varying annotation preferences, multiple clients may annotate the same data samples with different labels. For instance, in a task involving the perception of visual intent, an individual user may choose to label the same image differently.

**Feature distribution skew**: This is the case when each client’s distribution of the feature ci(x) differs from the distribution of ci(y|x). Client *j* prefers and uses the number 9 in bold, while client *i* likes the number 9 and writes it down in thin font on the MNIST dataset. The probability that the data distribution for client *j* will display the number 9 in bold is higher. But in the data distribution for client *i*, ci(x) is different because there is a higher probability that the number 9 will be typed in an extremely thin font. However, since ci(y|x) is constant, all of the typefaces are 9.

**Feature condition skew**: This indicates that although ci(y) = cj(y),the distribution of functions may differ amongst clients, i.e., ci(x|y)≠pj(x|y). Data properties: Clients may not completely overlap, and this is mostly connected to vertical FL, which is frequently utilised in medical applications. This is helpful if individual clients link regions, for instance, there are many Shiba Inus in the Japan region and numerous Husky samples in the Siberian region, but all of their tags are dog-related [115].

**Label noise skew**: This shows that the included noise label percentage varies for each client. Due to differences in input costs and levels of expertise, clients’ data labels differ significantly, producing data with different levels of label noise. When the participating clients have different architectures, the challenge becomes even more difficult because the decision boundaries are inconsistent.

**Sample noise skew**: This indicates that the quality of each client’s private data varies and that varying levels of sampling noise are inherently introduced during the data collection process. Due to differences in clients’ capacities for data synthesis and collection, data collected by different clients may contain noisy or redundant information, which can complicate and confuse communication between clients

**Quantity skew**: A significant variation in the quantity of distinct client data ci(x,y) is referred to as quantity skew. For instance, client *i* has 10,000,000 data samples, whereas client *j* has 100 data samples. In other words, ci(x,y) has significantly different data.

In conclusion, the data distribution across clients is non-IID, meaning that different clients may have different data sizes, labels, features, or quality. This can lead to poor performance, slow convergence, or divergence in the global model. To address this challenge, some possible solutions are: (i) **data augmentation** to generate synthetic data to increase the diversity and balance of the data across clients; (ii) **personalisation** to adapt the global model to the local data of each client using techniques such as fine-tuning, meta-learning, or multi-task learning; and (iii) **clustering** to group similar clients based on their data characteristics and training separate models for each cluster.

### 4.2. Device Heterogeneity

In a decentralised FL setting, client performance can vary. Client devices in federated networks have different computational capabilities, network connectivity, storage, and communication abilities. This variability is due to differences in hardware (CPU and memory), network connectivity (3G, 4G, 5G, and WiFi), and/or power (battery level). The training phase of FL can involve multiple devices from different products, the generations of the device, the manufacturer of the device, and the type of the device, resulting in a network of heterogeneous devices with varying computational abilities, memory sizes, and battery capacity. As a result, the training period can vary significantly across clients, and it is not effective to treat all participants equally. To achieve optimal training results, FL needs to consider heterogeneous hardware configurations [1,69,105,110,117]. In what follows, we introduce the device heterogeneity challenges and their implications on FL.

#### 4.2.1. Generation of the Device

The generation of a device refers to its release year or technological era. In FL, devices from different generations may have varying capabilities, processing power, memory capacity, and network connectivity. These differences can impact their performance and ability to participate in FL tasks. Newer generations of devices often come with more advanced hardware components, such as faster processors, larger memory capacities, and improved network capabilities. These advancements enable them to handle more complex ML tasks and contribute more effectively to FL models. On the other hand, older generations of devices may have limited resources and outdated hardware components. They might struggle with processing power or have lower memory capacities, which can affect their ability to perform computationally intensive tasks required in FL. However, it is important to note that even older devices can still contribute valuable data to FL models. If the system includes devices from different generations, such as older Android devices and newer iPhone devices, the data collected and transmitted may differ in terms of format, resolution, and quality. This can lead to issues in model training and convergence, as the system may struggle to handle the differences in data formats and quality.

Smartphones provide a clear example of heterogeneity based on the generation of the device. Newer generations of smartphones are equipped with more advanced hardware, including faster processors, larger memory, and enhanced ML capabilities. This hardware diversity can lead to the following issues and opportunities:

**Performance Variability**: Newer smartphones can execute complex ML tasks more quickly and efficiently compared to older models. For instance, running a deep learning model for image recognition may be significantly faster on a flagship smartphone released in 2023 than on a budget smartphone from 2018.

**Compatibility Challenges**: The ML framework used for FL must be compatible with a wide range of smartphone generations. Developers may need to optimise their algorithms to ensure that older devices can still participate effectively.

**Potential for Specialisation**: Newer smartphones may support hardware acceleration for specific ML operations, such as on-device AI chips. FL algorithms can take advantage of these capabilities, potentially offloading some computation to improve model training efficiency.

#### 4.2.2. Manufacturer of the Device

The manufacturer of a device plays a significant role in determining its heterogeneity in FL. Different manufacturers produce devices with varying specifications, architectures, and optimisation techniques. These differences can impact how devices handle FL tasks and interact with the overall system. Manufacturers often have their own proprietary technologies and optimisations that are specific to their devices. For example, some manufacturers may focus on optimising power consumption, while others prioritise performance or security features. These variations can result in different trade-offs between computational efficiency and accuracy during FL. Furthermore, manufacturers may also have different levels of support for ML frameworks or libraries used in FL. This can affect the ease of integration and compatibility of devices with FL systems. If the system includes devices from different manufacturers, such as Samsung and Apple, the data collected and transmitted may differ in terms of format, resolution, and quality. This can lead to issues in model training and convergence, as the system may struggle to handle the differences in data formats and quality. Devices from different manufacturers can exhibit heterogeneity due to variations in hardware architecture and software ecosystems. Let us consider the case of Android devices and iPhone operating system (iOS) devices:

**Hardware Differences**: Android devices are manufactured by a variety of companies, leading to diverse hardware configurations, while iOS devices are exclusively manufactured by Apple. This diversity can result in different processing capabilities and available memory.

**Software Ecosystem**: Android and iOS have distinct software ecosystems, each with its development tools, app stores, and APIs. This can affect the way FL applications are developed and deployed on these platforms.

**Privacy and Security**: iOS devices are known for their strict privacy and security policies, which may limit the extent to which FL can access and share data on the device. Android devices offer more flexibility but may have varying degrees of security enforcement based on the manufacturer and model.

#### 4.2.3. Type of the Device

The type of device refers to the category or form factor of the device participating in FL. This can include smartphones, tablets, laptops, IoT devices, edge servers, or even specialised hardware like accelerators or dedicated ML devices. Different types of devices have distinct characteristics and capabilities that influence their heterogeneity in FL. For example, smartphones and IoT devices typically have limited computational resources and battery life compared to laptops or edge servers. This limitation may require specific optimisation techniques to ensure efficient participation in FL tasks. Specialised hardware, such as accelerators or dedicated ML devices, may offer enhanced performance for certain ML workloads. However, their availability and compatibility with FL frameworks need to be considered when designing a heterogeneous FL system. Let us consider a scenario where a FL system is trained on data collected from wearable devices. If the system includes devices of different types, such as smartwatches and fitness trackers, the data collected and transmitted may differ in terms of format, resolution, and quality. This can lead to issues in model training and convergence, as the system may struggle to handle the differences in data formats and quality. Different types of devices can participate in FL, such as smartphones and IoT devices. This introduces heterogeneity in terms of computational capabilities, data collection, and communication:

**Computational Power**: Smartphones typically have more computational power than IoT devices, enabling them to perform more complex ML tasks. IoT devices may have limited processing capabilities, making it necessary to adapt the FL algorithm to accommodate these constraints.

**Data Collection and Transmission**: IoT devices are often resource-constrained and may have sporadic network connectivity. FL algorithms must be designed to handle these limitations while ensuring data synchronisation and model updates.

**Application-Specific Considerations**: The type of device also influences the choice of FL use cases. For example, IoT devices are commonly used in industrial settings for predictive maintenance, while smartphones are employed for applications like personalised recommendation systems.

For instance, suppose there are three devices that want to participate in FL: a smartphone, a tablet, and a laptop. Each device has its own data, model, network, and hardware characteristics, which can be summarised as follows, assuming a particular scenario:

**Smartphone**: The smartphone is a fourth-generation device from Samsung. It is a mobile device that can collect data from various sensors, such as a camera, microphone, GPS, and accelerometer. The data is non-IID and imbalanced, meaning that it does not follow the same distribution as the other devices and has different proportions of classes or labels. The smartphone has a small and shallow model, such as a convolutional neural network (CNN) with few layers and filters, to fit its limited memory and computation resources. The smartphone has a wireless and unstable network connection, which can vary depending on the signal strength, bandwidth, latency, and interference. The smartphone has a low and variable hardware capacity, which depends on the battery level, CPU usage, and temperature.

**Tablet**: The tablet is a fifth-generation device from Apple. It is a semi-mobile device that can collect data from some sensors, such as a camera, microphone, and touch screen. The data is moderately non-IID and balanced, meaning that it follows a similar but not identical distribution as the other devices and has roughly equal proportions of classes or labels. The tablet has a medium and moderate model, such as a recurrent neural network (RNN) with several layers and units, to balance its memory and computation resources. The tablet has a wireless and stable network connection, which can maintain consistent signal strength, bandwidth, latency, and interference. The tablet has a medium and stable hardware capacity, which does not vary much depending on the battery level, CPU usage, and temperature.

**Laptop**: The laptop is a sixth-generation device from Dell. It is a stationary device that can collect data from a few sensors, such as a keyboard, mouse, and webcam. The data is IID and balanced, meaning that it follows the same distribution as the other devices and has equal proportions of classes or labels. The laptop has a large and deep neural network model, such as a transformer network with many layers and attention heads, to exploit its abundant memory and computation resources. The laptop has a wired and stable network connection, which can guarantee high signal strength, bandwidth, latency, and interference. The laptop has a high and stable hardware capacity, which does not depend on the battery level, CPU usage, or temperature.

Let us illustrate heterogeneity in FL by using examples based on IoT and WSN devices. These devices are usually distributed, resource-constrained, and linked together via wireless networks. They can collect and process various types of data, such as images, audio, video, text, temperature, humidity, pressure, etc. Some examples of heterogeneous FL scenarios involving IoT and WSN devices are:

**Smart home**: In a smart home environment, different IoT devices, such as smart speakers, smart TVs, smart cameras, smart thermostats, smart lights, etc., can collaborate to learn a shared model for tasks such as voice recognition, face recognition, activity recognition, etc. However, these devices may have different data distributions, depending on the location, usage, and preference of the users. For example, the smart speaker in the living room may have more data on music and entertainment, while the smart camera in the bedroom may have more data on security and privacy. Moreover, these devices may have different model architectures, depending on the type and size of the data they process. For example, the smart TV may have a large and deep model for high-resolution video processing, while the smart light may have a small and shallow model for low-power control. Furthermore, these devices may have different network environments, depending on the wireless protocol, channel quality, and interference level they use. For example, the smart thermostat may have a stable and reliable network connection via Wi-Fi, while the smart camera may have an unstable and noisy network connection via Bluetooth. Additionally, these devices may have different hardware capacities, depending on the memory, computation, and battery resources they have. For example, the smart speaker may have a high and stable hardware capacity with a plug-in power supply, while the smart light may have a low and variable hardware capacity with a battery-powered supply.

**Smart city**: In a smart city scenario, different WSN devices, such as traffic cameras, air quality sensors, weather stations, etc., can cooperate to learn a shared model for tasks such as traffic management, pollution monitoring, weather forecasting, etc. However, these devices may have different data distributions depending on the geographic, temporal, and spatial factors that affect the data they collect. For example, the traffic camera in a busy intersection may have more data on congestion and accidents, while the air quality sensor in a remote park may have more data on freshness and cleanliness. Moreover, these devices may have different model architectures, depending on the complexity and diversity of the data they analyse. For example, the weather station may have a complex and heterogeneous model for multi-modal data fusion, while the air quality sensor may have a simple and homogeneous model for single-modal data processing. Furthermore, these devices may have different network environments, depending on the wireless technology, bandwidth, and latency they experience. For example, the traffic camera may have a high-speed and low-delay network connection via 5G, while the weather station may have a low-speed and high-delay network connection via LoRa. Additionally, these devices may have different hardware capacities, depending on the storage, communication, and energy resources they consume. For example, the air quality sensor may have a large and efficient hardware capacity with a solar-powered supply, while the traffic camera may have a small and inefficient hardware capacity with a battery-powered supply.

FL can improve computational power, data collection, and mobile device performance by reducing communication overhead, enhancing data privacy, and adapting to device heterogeneity. Some of the recent researchers who have contributed to improving the FL algorithms include [118,119,120]. The authors in [118] propose a spectrum allocation optimisation mechanism and a device selection method for enhancing FL over a wireless mobile network. The optimisation problem for the FL algorithm aims to minimise time delay by considering the energy constraints of local devices. The problem is divided into two sub-problems: spectrum allocation and device selection. An energy-efficient spectrum allocation optimisation method is proposed, minimising computation and transmission delay while meeting energy constraints. The K-means algorithm is trained using model weights, enhancing clustering performance and reducing training time. A weight divergence-based device selection method is proposed to overcome non-IID datasets. The proposed method outperforms other baseline approaches and achieves the fastest convergence in the FL framework. FedNAS [119,120] is a federated neural architecture search algorithm that can optimise the model architectures for different devices. It can improve the performance and efficiency of FL. One possible way to improve FedNAS is to combine it with the spectrum allocation optimisation and device selection methods proposed in [118]. The optimisation of spectrum allocation can minimise the time delay of FL while considering the energy consumption of individual devices. Additionally, device selection can enable FL to achieve faster convergence on non-IID datasets. By integrating these methods with FedNAS, FL can benefit from both the optimal model architectures and the optimal resource management. This can enhance the accuracy and efficiency of FL over a wireless mobile network. Some practical considerations for future implementations should include the following: (i) to design highly adaptive and robust algorithms that can handle non-IID and dynamic data distribution among the devices, (ii) to explore more efficient and secure encryption schemes that can protect the model parameters and gradients during the communication, and (iii) to incorporate more advanced machine learning techniques, such as meta-learning, transfer learning, and reinforcement learning, into FL.

In general, when clients have different hardware capabilities, such as computing power (CPU), memory, battery or storage, it results in device heterogeneity, which leads to resource constraints, computation bottlenecks, or energy consumption issues. To address this challenge, some potential solutions are:

- **Compression**: Reducing the size or complexity of the model or the communication using techniques such as quantisation, pruning, or sparsification.
- **Adaptation**: Adjusting the model or the communication based on the device conditions using techniques such as adaptive learning rate, adaptive aggregation, or adaptive communication.
- **Selection**: Choosing the most suitable or available devices for participation using techniques such as incentive mechanisms, reputation systems, or active learning.

### 4.3. Architectural Heterogeneity

The most suitable architecture for a specific FL application will depend on various factors, such as the type of device, data set size, and desired training time. FL also encounters challenges such as architectural heterogeneity, communication overhead, and scalability. In this section, we analyse the heterogeneity in FL when implemented using two-tier, three-tier, and mixed architectures, which distribute the training process across different devices.

#### 4.3.1. Two-Tier Architecture

A two-tier FL architecture consists of a server and clients. Clients communicate directly with the server, which aggregates model updates from the clients and sends the updated model back to them. Clients train the model on their local data and send updates to the server. This architecture is simple and easy to implement, but may have high communication costs, low scalability with a large number of clients, or limited network bandwidth. It is best suited for applications with homogeneous devices and data. The direct communication reduces latency, making it suitable for real-time applications. However, it may struggle with heterogeneous devices and data types, and some devices may become bottlenecks in a highly diverse network [121].

#### 4.3.2. Three-Tier Architecture

A three-tier FL architecture consists of three levels: the server, the edge nodes, and the clients. The server is responsible for aggregating the model updates from the edge nodes and sending the updated model back to the edge nodes. The edge nodes are responsible for aggregating the model updates from the clients and sending the model updates to the server. The clients are responsible for training the model on their local data and sending the model updates to the edge nodes. This architecture can reduce the communication cost and improve the scalability by introducing an intermediate level of edge nodes, which can act as local aggregators and coordinators for the clients. However, this architecture may introduce additional complexity and latency in the learning process [122].

#### 4.3.3. Mixed Architecture

A mixed FL architecture is a hybrid of the n-tier (n = 2, 3,...) architectures. It allows some clients to directly communicate with the server, while others communicate with the edge nodes. This architecture can adapt to the heterogeneity of the clients, such as their computation and communication capabilities, data distribution, and availability. It can also balance the trade-off between communication cost and learning performance by dynamically adjusting the communication pattern among the server, the edge nodes, and the clients [123].

To handle the heterogeneity of devices in FL, including different tier characteristics, careful strategies are needed to ensure successful collaboration and model convergence. Below is the list of some potential solutions:

##### Tiered Aggregation and Model Customisation

- Two-Tier and Three-Tier Devices: Design specialised aggregation mechanisms that cater to different tiers. For instance, a hierarchical aggregation approach could be employed, where intermediate-tier devices aggregate models before sending them to higher-tier devices or the central server.
- Mixed-Tier Devices: Implement adaptive algorithms that adjust aggregation strategies based on the characteristics of each device. Weighted averaging or differential learning rates can be used to incorporate updates from diverse devices effectively.

##### Model Compression and Adaptation

- Two-Tier and Three-Tier Devices: Employ model compression techniques (e.g., knowledge distillation, pruning) to reduce the complexity of models on lower-tier devices, allowing them to participate effectively despite resource constraints.
- Mixed-Tier Devices: Develop adaptive models that can adjust their complexity or architecture dynamically based on the capabilities of different devices in the federation.

##### Dynamic Learning Rate and Model Personalisation

- Two-Tier and Three-Tier Devices: Utilise differential learning rates or personalised updates for different tiers, allowing slower-learning or resource-constrained devices to adapt their models more gradually.
- Mixed-Tier Devices: Incorporate personalised learning strategies that cater to individual device capabilities, allowing for customisation of model updates based on the device’s resources and data characteristics.

##### Transfer Learning and Federated Meta-Learning

- Two-Tier and Three-Tier Devices: Implement transfer learning techniques that leverage knowledge from higher-tier devices to facilitate learning on lower-tier devices, enabling more efficient learning despite disparities in capabilities.
- Mixed-Tier Devices: Employ federated meta-learning approaches where models learn how to learn across devices of different tiers, allowing for adaptation and knowledge transfer between diverse devices.

##### Adaptive Communication and Resource Allocation

- Two-Tier and Three-Tier Devices: Develop adaptive communication protocols that prioritise communication and model updates based on the hierarchy of devices, optimising resource allocation.
- Mixed-Tier Devices: Implement resource-aware algorithms to dynamically allocate resources for model updates, allowing devices with varying capabilities to participate optimally without being constrained.

##### FL Simulators and Benchmarking

- All Tiers: Create FL simulators to test and benchmark algorithms across heterogeneous device tiers, enabling developers to assess performance and optimise algorithms under different scenarios.

### 4.4. Network and Communication Heterogeneity

FL encounters challenges when used in diverse networking and communication systems, including WiFi/cellular networks, WSNs, edge computing, and the IoT. These challenges arise from variations in data distributions among devices or nodes, which can affect the quality and convergence of the global model. Additionally, differences in model architectures and parameters among devices or nodes can impact the compatibility and communication efficiency of the global model. Variations in network environments and communication resources among devices or nodes can impact the reliability and latency of the global model. Lastly, differences in hardware devices and computational capabilities among the participating nodes can affect the performance of the global model and the energy consumption of the entire FL system [19,100,124].

#### 4.4.1. Network Heterogeneity

Network heterogeneity refers to the variation in the network characteristics of the devices participating in FL. This can include factors such as network speed, bandwidth, latency, and reliability. Network heterogeneity can impact the performance of FL in a number of ways. For example, devices with slow network connections may take longer to download and upload model updates, which can slow down the training process. Additionally, devices with unreliable networks may experience more errors during the training process [116] due to the delayed or failed packet reception.

#### 4.4.2. Communication Heterogeneity

Communication heterogeneity refers to the variation in the communication patterns of the devices participating in FL. This can include factors such as the number of devices participating in the training process, the frequency with which the devices communicate with the server, and the amount of data that is transferred between the devices and the server. Communication heterogeneity can impact the performance of FL in a number of ways. For example, if a large number of devices are participating in the training process, the server may become overloaded, which can slow down the training process. Additionally, if the devices communicate with the server at different frequencies, it can be difficult to coordinate the training process [125,126]. The limited number of devices that can communicate concurrently can also be challenging.

In general, here are some potential strategies to address the challenges of heterogeneity that arise in network and communication:

**Communication cost**: The communication cost refers to the amount of data or time required to transmit the model parameters or gradients across clients. Communication costs can vary depending on the communication protocol, the network bandwidth, the network latency, or the network reliability. Communication costs can affect the performance and efficiency of the global model, as well as the energy consumption and privacy of the clients. To address this challenge, some possible solutions are:

- **Compression**: Compression techniques can reduce the size or complexity of the model or the communication using methods such as quantisation, pruning, or sparsification. Compression techniques can lower the communication cost, but they may also introduce some errors or losses in the model or the communication.
- **Optimisation**: Optimisation techniques can minimise the communication cost or maximise the communication efficiency using methods such as gradient compression, gradient sparsification, or gradient quantisation. Optimisation techniques can improve the communication quality, but they may also require some trade-offs or assumptions in the model or the communication.

**Communication frequency**: The communication frequency refers to how often or when the clients communicate with the server or each other. Communication frequency can vary depending on the communication protocol, the network availability, the network stability, or the network congestion. Communication frequency can affect the convergence and robustness of the global model, as well as the synchronisation and coordination of the clients. To address this challenge, some possible solutions are:

- **Synchronisation**: Synchronisation techniques can coordinate the communication frequency or timing using methods such as synchronous updates, asynchronous updates, or periodic updates. Synchronisation techniques can ensure the consistency and reliability of the global model, but they may also introduce some delays or overheads in the communication.
- **Adaptation**: Adaptation techniques can adjust the communication frequency or timing based on the network conditions or the client preferences using methods such as adaptive learning rate, adaptive aggregation, or adaptive communication. Adaptation techniques can enhance the flexibility and responsiveness of the global model, but they may also require some feedback or monitoring in the communication.

**Communication robustness**: Communication robustness refers to how well the communication can handle the errors or failures that may occur in the network or the clients. Communication robustness can vary depending on the communication protocol, the network reliability, the network security, or the network diversity. Communication robustness can affect the accuracy and stability of the global model, as well as the fault tolerance and resilience of the clients. To address this challenge, some possible solutions are:

- **Error correction**: Error-correction techniques can detect and correct the errors or losses that may occur in the model or the communication using methods such as checksums, parity bits, or error-correcting codes. Error-correction techniques can improve communication quality, but they may also increase communication costs or complexity.
- **Recovery mechanisms**: Recovery mechanisms can recover or restore the model or the communication from the failures or attacks that may occur in the network or the clients using methods such as checkpoints, backups, or replication. Recovery mechanisms can improve communication reliability, but they may also consume some resources or storage.

### 4.5. Model Heterogeneity

FL requires each client to use a local model with the same architecture, and then it aggregates the received updates into a global model. In IoT applications, clients may design unique local models due to individual requirements and hardware constraints. Model heterogeneity requires learning knowledge without sharing private data and model structure information. In such setting, transferring knowledge between heterogeneous clients is challenging. Partial heterogeneity refers to the case where certain clients utilise the same model structure while others do not. Partially heterogeneous models are those in which a federated system is thought to have. FL models are used to train each isomorphic client subset. Intra-cluster models can be aggregated using methods such as weighted averaging, but inter-cluster models require knowledge distillation. When participant models’ network structures vary within a FL framework, this is known as complete heterogeneity, a kind of partial heterogeneity. For every client, this produces a unique model, which could result in high learning overheads and ineffective communication [115]. Following are a few potential solutions to this model heterogeneity challenge:

**Alignment**: Making the model architectures compatible or consistent using techniques such as model conversion, model alignment, or model standardisation.

**Generation**: Creating the model weights or architectures dynamically using techniques such as hypernetworks, neural architecture search, or meta-learning.

**Evaluation**: Measuring the model’s performance or quality using techniques such as federated evaluation, federated testing, or federated validation.

### 4.6. Lessons Learnt

In general, the heterogeneity challenge in FL is a significant obstacle, involving various aspects such as statistical, device, architectural, network and communication, and model heterogeneity. These hurdles require tailored solutions to mitigate their impact on FL performance. The challenge stems from the inherent diversity in the FL landscape, which can lead to suboptimal performance, compromised model accuracy, and inefficient collaboration among devices. To overcome this, the chapter provides a comprehensive and systematic analysis of the heterogeneity challenge in FL and proposes a taxonomy of solutions for each category of heterogeneity, such as data augmentation, personalisation, clustering, adaptive learning rate, gradient compression, device selection, hierarchical aggregation, edge computing, hybrid communication, asynchronous updates, and more.

In light of these insights, the next steps involve implementing some of the aforementioned solutions, refining them, and continuously monitoring their efficacy. A holistic framework is needed to overcome the heterogeneity challenge in FL and foster collaborative learning across diverse environments, and this chapter can also serve as a valuable reference for researchers and practitioners who are interested in addressing the heterogeneity challenge in FL and enhancing the performance and applicability of FL in various scenarios.

Careful system design and continuous system maintenance can help avoid certain types of heterogeneity. For example, instead of allowing ad hoc formation of the FL system, the system manager may constantly reform the system in such a way that a particular system architecture is maintained. In this manner, the FL system may not encounter architectural heterogeneity. On the other hand, certain types of heterogeneity will emerge soon or later. For example, it is not practical to assume that all FL client devices are homogeneous and their data distributions are perfectly IID. Luckily, many aspects of heterogeneity are widely studied, and there are efficient solutions that minimise performance degradation. Also, by carefully analysing the given FL system, the particular intelligent application(s) running on the system, and the characteristics of the dataset, a certain level of heterogeneity of a particular type can be ignored. For example, considering the fact that smartphones nowadays are getting richer in terms of computing power and storage and that common network standards allow different devices to communicate with each other, a certain level of device heterogeneity may not cause any noticeable effect on the FL system.

## 5. Security and Privacy Considerations

Security and privacy concerns are one of the major issues in the field of IoT, WSNs, and FL. These technologies involve the collection, processing, and sharing of vast amounts of data, making them vulnerable to various threats. In this section, we address these concerns and discuss threats and vulnerabilities that can compromise data confidentiality, integrity, and availability associated with IoT and WSNs, as well as the convergence of the FL model. We also explore different techniques and propose solutions for ensuring model convergence, data confidentiality, integrity, and availability in IoT, WSNs, and FL systems.

### 5.1. Threats and Vulnerabilities

#### 5.1.1. Unauthorised Access

One of the primary concerns in IoT, WSNs, and FL is unauthorised access to devices or networks. Attackers may exploit vulnerabilities in the system to gain unauthorised access and control over IoT devices or sensor nodes.Unauthorised access to the training data could lead to the exposure of sensitive information, such as personal data or proprietary information. This can lead to various malicious activities such as data theft, device manipulation, or even physical harm [49,127].

#### 5.1.2. Data Breaches

The vast amount of data generated by IoT devices and WSNs makes them attractive targets for data breaches. If proper security measures are not in place, attackers can intercept or manipulate the data during transmission or storage. This can result in the exposure of sensitive information or the compromise of system integrityThere are several factors that contribute to the risk of data breaches in FL. These include gradient information leakage, non-IID data distribution, and a lack of robust security protocols. Gradient information leakage occurs when the exchange of gradient updates during FL training reveals information about the underlying data. Non-IID data distribution means that FL often involves training on non-identically distributed data, making it more susceptible to inference attacks. Additionally, FL systems may lack adequate security measures to protect against data breaches [22].

#### 5.1.3. Denial-of-Service (DoS) Attack

IoT systems and WSNs are vulnerable to DoS attacks, in which attackers flood the network with a high volume of requests or malicious traffic. This can overwhelm the system and make it unavailable for legitimate users, disrupting critical services or rendering the entire system non-functional. DoS attacks can also overwhelm the FL server, consuming resources, preventing user access, exposing sensitive data, and compromising privacy, leading to inaccurate or unreliable models [128].

#### 5.1.4. Malware and Botnets

Malware and botnets pose a threat to the integrity and availability of FL systems by infecting IoT devices and launching malicious attacks. Malware refers to any software intentionally designed to cause damage or harm to a computer, network, server, or client. Botnets are networks of computers infected by malware and controlled by a single attacking party, known as the bot-herder. Botnets can engage in various malicious activities, including sending spam, launching distributed denial-of-service (DDoS) attacks, generating fake Internet traffic, and stealing sensitive information. Another concern is model stealing, where malicious participants attempt to infer or reconstruct the private data or model parameters of other participants by observing the global model or the model updates. This violates the data privacy and security of the FL system and its participants [129,130,131].

#### 5.1.5. Physical Attacks

Physical attacks in FL target the hardware or devices of the participants, such as tampering, stealing, or destroying them. These attacks can result in data loss, model corruption, or privacy leakage. For instance, an attacker could physically access a device to extract its local data or model parameters, or modify them to inject malicious behavior into the global model. Physical attacks can also impact the availability and reliability of the FL system, as some devices may become unavailable or unresponsive due to damage or theft. Therefore, it is crucial to protect the devices and hardware involved in FL from physical attacks [132].

#### 5.1.6. Poisoning Attack

A poisoning attack in FL refers to a malicious act where participants in a distributed learning system attempt to compromise the global model by sending corrupted updates to the server. There are various methods and goals associated with poisoning attacks, including backdoor attacks, label-flipping attacks, and targeted attacks. A poisoning attack allows an attacker to manipulate a portion of the training data by assigning attacking labels. This manipulation changes the model parameters of the target learning model during the training phase. As a result, the poisoned learning model will exhibit certain properties desired by the attacker, causing the misclassification of selected inputs during the inference stage [35,133,134,135].

#### 5.1.7. Byzantine Attack

A Byzantine attack is a type of attack in a distributed system where machines upload malicious data instead of legitimate computational output. This attack specifically targets user collusion in a distributed learning environment, such as FL. Byzantine users, or multiple clients, can be controlled by a malicious attacker in FL. These users may upload fraudulent data due to malicious attacks, faulty hardware, or unreliable communication channels. The attacker’s manipulation can distort the global model and prevent it from converging. As a result, these malicious models can severely hinder the training process and the aggregation of the global model [136,137].

### 5.2. Techniques for Ensuring Data Confidentiality, Integrity, and Availability

#### 5.2.1. Encryption

Encryption is a fundamental technique for ensuring data confidentiality in IoT systems. By encrypting the data during transmission and storage, even if an attacker intercepts it, it will not be able to decipher the information without the encryption key [138].

#### 5.2.2. Access Control

Implementing robust access control mechanisms is crucial for preventing unauthorised access to IoT devices or networks. This involves authentication and authorisation processes to ensure that only authorised individuals or entities can access and control the devices or networks [8,127,139].

#### 5.2.3. Intrusion Detection and Prevention Systems (IDPS)

IDPS can help detect and prevent various types of attacks in IoT systems. These systems monitor network traffic, analyse patterns, and identify any suspicious activities or anomalies that may indicate an ongoing attack. They can then take proactive measures to mitigate the attack and protect the system [49].

#### 5.2.4. Secure Communication Protocols

Using secure communication protocols such as Transport Layer Security (TLS) or Secure Shell (SSH) can ensure the integrity and confidentiality of data transmitted between IoT devices or sensor nodes. These protocols provide encryption, authentication, and data integrity checks to prevent eavesdropping, tampering, or spoofing attacks [4].

#### 5.2.5. Regular Updates and Patch Management

Keeping IoT devices, WSNs, and FL frameworks up-to-date with the latest security patches is essential for mitigating vulnerabilities. Regular updates help address known security issues and protect against emerging threats [7,49].

#### 5.2.6. Physical Security Measures

Implementing physical security measures such as tamper-proof seals, secure enclosures, or biometric access controls can help prevent physical attacks on IoT devices or sensor nodes. Using secure hardware modules or trusted execution environments to isolate the computation and communication of FL from other applications or processes on the device, and to prevent unauthorised access or tampering. Implementing authentication and authorisation mechanisms to verify the identity and legitimacy of the devices and parties involved in FL, and to reject any unauthorised or suspicious requests or updates [140,141].

#### 5.2.7. Privacy-Preserving Techniques

Privacy-preserving techniques such as data anonymisation, pseudonymisation, or differential privacy can be employed to protect the privacy of individuals whose data is collected by IoT devices or WSNs. These techniques ensure that sensitive information cannot be directly linked to specific individuals as discussed in the below [20,67,68,111,112].

##### Secure Multi-Party Computing

Secure multi-party computing (SMC) is a cryptographic technique that allows multiple parties to jointly compute a function or a value without revealing their inputs to each other. SMC can be used to enhance the privacy and security of FL. The parties retain total control over the data they own throughout the computation process, knowing nothing more than their individual inputs and outputs [142].

##### Differential Privacy

Differential privacy (DP) technology uses random noise to drown original data, preventing attackers from reversing it. Implemented by adding noise to a query, DP protects computational results, is independent of background knowledge, and can theoretically resist attacks [143].

##### Homomorphic Encryption

In FL systems, homomorphic encryption (HE) is a privacy-preserving cryptographic technique that enables certain computations on encrypted data without the need to first decrypt it. HE types include partial, fully, and somewhat HE, providing security for cross-silo FL by performing complex computation operations [144].

The choice of solution depends on specific use cases, regulatory requirements, and trade-offs between security, privacy, feasibility, efficiency, and practicality. Organisations may choose a combination of these solutions to effectively address heterogeneity, security, and privacy concerns.

##### Heterogeneity

**Solution 1: Standardisation**

Feasibility: Standardisation involves implementing uniform protocols, which can be challenging due to the diverse systems and technologies in use. However, initiatives such as industry-wide consortiums or the adoption of widely accepted protocols (e.g., HTTP for web communication) could enhance feasibility.

Efficiency: Standardisation streamlines communication but may stifle innovation or impede system-specific optimisations.

Practicality: Achieving full standardisation across diverse platforms may not be feasible due to entrenched systems and varying requirements, although it may be achievable in specific industries or regions.

**Solution 2: Middleware**

Feasibility: Implementing middleware can bridge the gap between heterogeneous systems, facilitating effective communication between them. However, integrating multiple technologies into a unified middleware solution can be complex.

Efficiency: Middleware can improve efficiency by offering a standardised interface, but it may also introduce latency or compatibility issues.

Practicality: Middleware is advantageous for specific use cases that require integration but may not be suitable for highly specialised systems or applications.

##### Security

**Solution 1: Encryption and Authentication Protocols**

Feasibility: Implementing robust encryption and authentication protocols is feasible, but it may require updates to address evolving threats.

Efficiency: Strong encryption can slow down processes, impacting efficiency, but it enhances security.

Practicality: This approach is feasible for securing data transmission and storage, but it requires ongoing updates and maintenance to stay ahead of vulnerabilities.

**Solution 2: Multi-factor Authentication (MFA)**

Feasibility: Implementing MFA is achievable with modern authentication frameworks, but it may require user training and behavioral adjustments.

Efficiency: Enhancing security measures may result in minor delays in the user access process.

Practicality: This method is effective for securing access to sensitive systems, especially where data security is paramount. However, users may resist the implementation.

##### Privacy

**Solution 1: Data Minimisation and Anonymisation**

Feasibility: Minimising and anonymising data is possible, but it may require significant effort to redesign systems and processes.

Efficiency: Improving privacy measures may result in a decrease in the depth of data analysis or its usefulness in certain situations.

Practicality: It is feasible to comply with privacy regulations and protect user data, but it may pose challenges for data-driven features that rely on extensive data analysis.

**Solution 2: Differential Privacy Techniques**

Feasibility: Implementing differential privacy can be complex, requiring expertise and careful implementation.

Efficiency: It effectively preserves individual privacy but may impact the accuracy of aggregate data analysis.

Practicality: This approach is suitable for situations where ensuring individual privacy is crucial. However, it may be necessary to strike a balance between accuracy and privacy protection.

##### Comparative Analysis

Standardisation vs. Middleware: Standardisation can promote uniformity but may stifle innovation, while middleware offers flexibility but can also introduce complexity.

Encryption vs. MFA: Encryption secures data transmission and storage, while MFA secures access points, offering layered security.

Data Minimisation vs. Differential Privacy: Minimisation ensures less data exposure, while differential privacy preserves individual privacy at the expense of some accuracy.

In conclusion, security and privacy concerns are significant challenges in IoT, WSNs, and FL. Unauthorised access, data breaches, DoS attacks, malware, physical attacks, and other threats pose risks to the confidentiality, integrity, and availability of data in these systems. However, by implementing techniques such as encryption, access control, IDPS, secure communication protocols, regular updates, physical security measures, and privacy-preserving techniques, it is possible to mitigate these risks and ensure the security and privacy of IoT systems by using features of FL. Some instances where FL significantly outperforms other methods for preserving privacy while training models across distributed IoT devices are as follows: FL can use generative adversarial networks (GANs) to generate synthetic data that preserves the statistical properties of the real data but does not reveal any sensitive information [145]. FL can also use secret sharing or split learning to divide the model or data into multiple parts and distribute them among different parties so that no single party can access the complete model or data. Additionally, FL can be integrated with the blockchain to achieve decentralised and secure learning without relying on a central server or authority. Blockchain can ensure data integrity and prevent single-point failure and poisoning attacks by using cryptographic techniques and consensus mechanisms [12]. Blockchain can also provide a personalised incentive mechanism for clients to participate in FL [146,147].

### 5.3. Lessons Learnt

In general, security and privacy concerns emerge large in the domains of IoT, WSNs, and FL, where the extensive collection, processing, and sharing of data expose these technologies to a variety of threats and vulnerabilities. This section delves into the details of these concerns, highlighting potential risks associated with unauthorised access, data breaches, denial-of-service attacks, malware, physical attacks, and more. The exploration encompasses the imperative need for robust security measures in the convergence of FL models within the interconnected IoT and WSN frameworks.

The chapter unfolds with a comprehensive understanding of threats and vulnerabilities, ranging from unauthorised access to Byzantine attacks. It then delves into techniques for ensuring data confidentiality, integrity, and availability, covering encryption, access control, intrusion-detection systems, secure communication protocols, and privacy-preserving techniques such as homomorphic encryption. Solutions proposed to address these security and privacy challenges involve standardisation, middleware, encryption and authentication protocols, multi-factor authentication, and privacy techniques such as data minimisation and anonymisation. The section concludes with a comparative analysis of these proposed solutions, offering insights into their effectiveness in mitigating the identified threats and vulnerabilities. It also provides a comprehensive and systematic overview of the security and privacy issues in IoT, WSNs, and FL, presents a taxonomy of techniques and solutions for addressing these issues, and can serve as a useful guide for researchers and practitioners who are interested in developing secure and privacy-preserving IoT systems and FL applications.

It is worth noting that security threats can also affect the FL system’s performance. In a general FL system, each trained model from the FL clients contributes to the completion of the global model. That means if a client’s model is trained on a compromised dataset or if the uploaded model is altered in an undesirable way, the global model may suffer from low accuracy, delayed convergence, or even divergence. One may take different approaches to tackle such threats than conventional security-related solutions. For example, anomaly detection can be used to identify outlier models so that they do not participate in the global model aggregation. Also, one may compare the models among the devices that are expected to accumulate data samples from similar distributions to identify suspicious FL clients. In sum, the security expert of a FL system should also pay attention to the aspects that can affect the performance of the global models.

In addition, although FL can protect privacy by not uploading the dataset collected by FL clients, it still requires FL clients to upload the trained model, meaning that the structure of the model can be exposed to the eavesdropper. As a solution to the model exposure issues, split learning [148] can be a solution, which is out of the scope of this paper.

## 6. Performance Evaluation

Performance evaluation methodologies play a crucial role in assessing the effectiveness and efficiency of systems related to the IoT, WSNs, and FL. These methodologies involve the use of various metrics to measure system performance, including latency, energy consumption, scalability, accuracy, and communication overhead. By evaluating these metrics, researchers can gain insights into the strengths and weaknesses of different systems and make informed decisions regarding their design and optimisation [47,48,79,83].

Latency is a key metric used to evaluate the performance of IoT, WSNs, and FL systems. It refers to the time delay between the initiation of a request or task and the corresponding response or completion. In IoT applications, low latency is often critical for real-time monitoring and control tasks. For example, in industrial IoT applications, minimising latency is essential for ensuring timely responses to critical events. In WSNs, latency affects the timeliness of data delivery from sensor nodes to the sink node or base station. Similarly, in FL systems, latency impacts the speed at which model updates are propagated among participating devices.

Energy consumption is another important metric in evaluating system performance. In IoT deployments, devices are often battery-powered or have limited energy resources. Therefore, minimising energy consumption is crucial for prolonging device lifetime and reducing maintenance costs. In WSNs, where sensor nodes are typically deployed in large numbers and may be difficult to access for battery replacement, energy efficiency is paramount. FL systems also need to consider energy consumption as participating devices may have limited power resources [48,79].

Scalability is a metric that measures how well a system can handle increasing workloads or accommodate a growing number of devices or users. In IoT applications, scalability is crucial as the number of connected devices can range from a few to billions. Scalable systems can handle this growth without a significant degradation in performance or resource utilisation. Similarly, in WSNs, scalability is essential to support large-scale deployments and ensure efficient data collection. FL systems also need to be scalable to accommodate a large number of participating devices and handle increasing model sizes. Accuracy is a metric used to evaluate the correctness of system outputs or predictions. In IoT applications, accuracy is crucial for tasks such as anomaly detection, predictive maintenance, and decision making based on sensor data. In WSNs, accuracy is important for ensuring reliable and trustworthy data collection. FL systems also need to maintain high accuracy levels to ensure the quality of the aggregated models.

Communication overhead refers to the additional resources consumed by communication processes in a system. This metric includes factors such as bandwidth utilisation, message size, and network congestion. In IoT applications, minimising communication overhead is essential for efficient use of network resources and reducing latency. In WSNs, communication overhead affects energy consumption and network capacity. FL systems also need to consider communication overhead as it impacts the time and energy required for model updates and aggregation [4].

To evaluate these performance metrics, researchers employ various methodologies in their studies related to IoT, WSNs, and FL. These methodologies often involve simulation-based approaches, testbed experiments, or analytical models. Simulations allow researchers to assess system performance under different scenarios and conditions while providing control over various parameters. Testbed experiments involve deploying real hardware and software components in a controlled environment to evaluate system performance under realistic conditions. Analytical models provide theoretical insights into system behavior and performance characteristics.

In [149], the authors provide a comprehensive review of the recent advances in FL methods for medical image analysis and also present some experimental results to compare the performance of FL and centralised learning on different medical imaging tasks. One of the tasks is the classification of brain tumour subtypes using magnetic resonance imaging (MRI) data from multiple hospitals. The paper reports that by using FL with only 10 epochs for model updates, the average accuracy of the classification model was improved from 75% to 90.8%, compared to centralised learning with 50 epochs. This shows that FL can achieve significant improvements in diagnostic accuracy (up to 15%) compared to traditional centralised methods with fewer training iterations while also preserving the privacy and security of hospital data. This illustrates the effectiveness of FL despite fewer training iterations. In [150], the authors report that by using FL and an asynchronous graph convolutional network, the average accuracy of the prediction model was improved by up to approximately 6.85% in Root Mean Squared Error and 20.45% in Mean Absolute Percentage Error compared to the existing models. This shows that FL can achieve comparable or even better accuracy with fewer epochs while also reducing the communication and computation overheads of centralised learning.

In [151], the authors introduce an approach to end-to-end on-device machine learning by utilising FL and validate it with wheel steering angle prediction for autonomous driving vehicles. The model decreases training time by 75% and bandwidth costs by 25% while achieving the same level of prediction accuracy as the widely used centralised learning method. In [152], the authors proposed a FL framework for IoT devices, utilising DT and edge networks for reliable real-time data processing. The framework increases data privacy, enhances system security, and reduces latency. The Deep Reinforcement Learning (Deep-RL) agent optimises resource allocation and energy consumption, ensuring real-time data-processing interactions between IoT devices and edge servers. The Deep-RL-agent-based DT optimises bandwidth allocation, localisation, and transmission costs, enhancing learning efficiency, and the approach effectively selects 47.5% of local computing activities with 1 MHz bandwidth, thereby minimising the weighted cost of edge-computing strategies.

In [153], the authors propose an efficient adaptive algorithm called FAFED based on the momentum-based variance-reduced technique in cross-silo FL. The authors describe the architecture of FAFED and its key components, including the edge server, the edge device, and the cloud server. The paper shows that FAFED is the first adaptive FL algorithm to achieve the best-known sample complexity of $O(\epsilon^{-3})$ and $O(\epsilon^{-2})$ communication rounds for finding an ϵ-stationary point without using large batches. The experimental results on the language modelling task and the image classification task with heterogeneous data demonstrate the efficiency of FAFED. For the computational complexity of FL, this work is an invaluable resource.

In conclusion, performance evaluation methodologies play a crucial role in assessing the effectiveness and efficiency of IoT, WSNs, and FL systems. Metrics such as latency, energy consumption, scalability, accuracy, and communication overhead are used to measure system performance. By evaluating these metrics through simulation-based approaches, testbed experiments, or analytical models, researchers can gain valuable insights into system behavior and make informed decisions regarding system design and optimisation.

## 7. Future Directions and Vision

In our visionary pursuit, we aspire to establish a cutting-edge paradigm that facilitates secure and privacy-preserving data collaboration across heterogeneous IoT and WSNs through the implementation of FL. Our overarching goal is to craft a unified framework that seamlessly integrates the realms of IoT, wireless sensor networks, and federated learning, meticulously addressing the inherent challenges associated with data heterogeneity, security, and privacy. By leveraging the capabilities of FL, we intend to overcome the intricacies presented by diverse data sources, ensuring a harmonised approach that not only upholds the integrity of the integrated system but also safeguards against potential security threats and privacy breaches. This comprehensive vision demonstrates our commitment to advancing the understanding and practical application of a unified ecosystem in which the synergy between IoT, WSNs, and FL not only thrives but also sets a benchmark for secure, privacy-preserving data collaboration in the ever-changing landscape of technological integration. By prioritising these key elements, our objective is to maintain a lucid and cohesive narrative that not only adds depth but significantly contributes to a broader understanding of the subject matter.

Additionally, we aim to enrich our exploration by incorporating findings related to performance measurement metrics, including latency, energy consumption, scalability, accuracy, and communication overhead, ensuring that only the most pertinent insights are integrated. Through this strategic approach, our vision is to elevate the overall quality and impact of our article, providing a comprehensive and insightful resource for those seeking a deeper understanding of the integration of IoT, WSNS, and FL. This paper’s relevant views are summarised as follows:

- To enable secure and privacy-preserving data collaboration across heterogeneous IoT and WSNs using FL.
- To create a unified framework for integrating IoT, WSNs, and FL that respects data heterogeneity, security, and privacy.
- To leverage FL to overcome the challenges of heterogeneity, security, and privacy in IoT and WSN integration.

The existing literature on the integration of IoT, WSNs, and FL has made significant progress in understanding the potential benefits and challenges of combining these technologies. However, there are still several gaps in the current research that need to be addressed, and future studies can explore various directions to further enhance the integration of IoT, WSNs, and FL.

### 7.1. Standardisation of Protocols

One of the main gaps in the existing literature is the lack of standardised protocols and frameworks for integrating IoT, WSNs, and FL. While there have been efforts to develop protocols such as MQTT and CoAP for IoT communication, there is a need for similar standards specifically tailored for WSNs and FL. Standardisation would enable interoperability between different devices and systems, facilitating seamless integration and collaboration.

### 7.2. Security and Privacy Considerations

Another gap in the literature is the limited understanding of the security and privacy implications of integrating IoT, WSNs, and FL. As these technologies involve collecting and analysing sensitive data from various sources, ensuring data confidentiality, integrity, and privacy becomes crucial. Future research should focus on developing robust security mechanisms that can protect data during transmission, storage, and processing. Additionally, privacy-preserving techniques such as differential privacy can be explored to mitigate the risk of re-identification attacks.

### 7.3. Scalability Challenges

Furthermore, there is a need for more comprehensive studies on the scalability and resource constraints associated with integrating IoT, WSNs, and FL. As the number of connected devices continues to grow exponentially, scalability becomes a critical factor. Researchers should investigate how to efficiently handle large-scale deployments of IoT devices and WSNs while maintaining low latency and high throughput. Moreover, considering the resource-constrained nature of many IoT devices and WSNs, energy-efficient algorithms and optimisation techniques should be developed to minimise power consumption without compromising performance.

### 7.4. Edge and Fog Computing Paradigms

In terms of future research directions, one promising area is the exploration of edge computing and fog computing paradigms in the integration of IoT, WSNs, and FL. Edge computing involves processing data closer to the source, reducing latency and bandwidth requirements. Fog computing extends this concept by distributing computing resources across multiple edge devices, enabling more efficient data processing and analysis. By leveraging these paradigms, researchers can investigate how to offload computationally intensive FL tasks to edge devices or fog nodes, improving overall system performance.

### 7.5. Adaptive and Self-Organising Algorithms

Another potential research direction is the development of adaptive and self-organising algorithms for IoT, WSNs, and FL integration. Traditional centralised approaches may not be suitable for dynamic and heterogeneous environments where IoT devices and WSNs can join or leave the network at any time. Adaptive algorithms can dynamically adjust their behavior based on changing network conditions, ensuring robustness and scalability. Self-organising algorithms can enable autonomous coordination and collaboration among distributed devices, optimising resource utilisation and enhancing system resilience.

### 7.6. Integration of AI Techniques

Additionally, future research can explore the integration of artificial intelligence (AI) techniques such as ML and DL with IoT, WSNs, and FL. AI algorithms can enhance the capabilities of IoT devices and WSNs by enabling intelligent decision-making, anomaly detection, and predictive analytics. By combining AI with FL, researchers can investigate how to train models collaboratively on distributed data while preserving privacy, enabling more accurate and efficient analysis.

In conclusion, while the existing literature has made significant progress in understanding the integration of IoT, WSNs, and FL, there are still gaps that need to be addressed. The standardisation of protocols, security and privacy considerations, scalability challenges, edge computing and fog computing paradigms, adaptive and self-organising algorithms, and the integration of AI techniques are all potential areas for future research. By exploring these directions, researchers can further enhance the integration of IoT, WSNs, and FL, unlocking their full potential in various domains.

## 8. Conclusions

This systematic literature review conducted on the integration of IoT, WSNs, and FL has revealed several key findings. This review aimed to explore the current state of research in this area and identify the potential benefits and challenges associated with integrating the three core technologies to enable intelligent decision-making in various domains.

Firstly, the review found that the integration of IoT, WSNs, and FL has the potential to revolutionise data collection, data exchange, and decision-making processes across different domains. By leveraging the vast amount of data collected by IoT devices and WSNs, FL algorithms can be used to train models collaboratively without sharing raw data. This enables organisations to make more accurate and informed decisions based on real-time data analysis.

Secondly, the review highlighted the importance of data privacy and security when integrating these technologies. As IoT devices and WSNs may collect sensitive data, ensuring the privacy and security of this data becomes crucial. FL provides a solution to this challenge by allowing model training without exposing raw data, thereby preserving privacy while still benefiting from collective intelligence.

Additionally, the review identified several domains where the integration of IoT, WSNs, and FL can have significant impacts. These domains include healthcare, transportation, agriculture, smart cities, industrial automation, and environmental monitoring. In healthcare, for example, integrating these technologies can enable remote patient monitoring, early disease detection, and personalised treatment plans. In transportation, it can facilitate intelligent traffic management systems and autonomous vehicles. In agriculture, it can optimise irrigation systems and crop yield prediction. These examples demonstrate the wide-ranging applications and potential benefits of integrating IoT, WSNs, and FL.

Heterogeneity in FL can have a significant impact on the accuracy and reliability of the model to be trained in a distributed manner. The statistical, architecture, network, communication, model, generation of the device, manufacturer of the device, and type of device can all contribute to the issue of heterogeneity. To address this challenge, it is essential to develop sophisticated methods and techniques that can handle the variations in data formats, quality, and capabilities of devices in a FL system.

Furthermore, the review emphasised the need for further research in this area. While there are already numerous studies exploring different aspects of integrating IoT, WSNs, and FL, there is still much to be explored in terms of scalability, interoperability, energy efficiency, and algorithm optimisation. Future research should focus on addressing these challenges to fully realise the potential of this integration.

In conclusion, this systematic literature review highlights the importance of integrating IoT, WSNs, and FL for enabling intelligent decision making in various domains. The review reveals that this integration has the potential to revolutionise decision-making processes, preserve data privacy and security, and have significant impacts in domains such as healthcare, transportation, agriculture, smart cities, industrial automation, and environmental monitoring. However, further research is needed to address scalability, interoperability, energy efficiency, and algorithm optimisation challenges. 

## Figures and Tables

**Table 1:** A summary of state-of-the-art research in FL heterogeneity classified by H (Heterogeneity), S (Security or privacy), and O (Other systems assisting FL such as IoT and WSN.), where ✓, △, and ✗ indicates comprehensive, partial, and little coverage, respectively.

Research (Year)	Key Concept			Main Findings	Limitation
	**H**	**S**	**O**		
[102] (2022)	△	✗	✓	Present impact of heterogeneity causing significant degradation in performance, fairness, and test accuracy in models trained in FL compared to uniform settings.	Strategies for addressing heterogeneity are not provided, and analysis on FedAvg, FedProx, and Q-FFL is limited.
[103] (2019)	△	✓	✓	Highlights case studies and data security issues while discussing the many components of the FL system, e.g., data distribution, ML models, privacy protections, and communication architecture.	Does not effectively address the issue of heterogeneity.
[1] (2022)	△	✓	✓	Survey of FL for the management of resources of IoT networking system with a possible solution and previous limitations. Importance of FL for IoT-based devices with limited resources.	Security attacks in IoT networking systems are a major concern that requires thorough discussion. Furthermore, the issue of heterogeneity has not been effectively addressed.
[13] (2022)	△	✓	✓	Explores the use of FL in IoT networks, addressing issues such as communication cost, robustness, and privacy, while also highlighting challenges and taxonomies.	Inadequate categorisation and examination of the difficulties posed by heterogeneity in FL.
[104] (2020)	△	✓	✓	Emphasises the importance of reducing communication overhead, addressing statistical and structural heterogeneity, and enhancing privacy within the FL framework. It also highlights the significance of incentive mechanisms, detecting malicious participants, secure aggregation, and protection methods.	Outlined the statistical and structural heterogeneity in FL without providing a comprehensive classification and synopsis of current approaches.
[5] (2021)	✗	✓	✓	Examine possible privacy leakage issues in FL and improve knowledge about privacy-preserving FL.	Heterogeneity issues were not discussed.
[35] (2021)	△	✓	✓	Covers recent developments and a general overview of FL applications and security concerns in multiple domains.	Does not effectively address the issue of heterogeneity.
[69] (2019)	△	✓	✓	They analyse the difficulties of FL from the perspectives of efficiency, heterogeneity, and privacy, and outline some potential approaches for the future.	Does not provide a comprehensive detailed classification and discussion of the challenges of heterogeneity.
[12] (2021)	△	✓	✓	The applications of FL in IoT networks are surveyed and examined.	Instead of addressing all potential FL scenarios, this work concentrates on the characteristics and requirements of IoT networks.
[105] (2022)	△	✓	✓	Provides an overview of FL, including its technologies, architectures, system issues, privacy-preserving techniques and applications. It also explores current and anticipated technological trends.	The heterogeneity problem was not effectively addressed.
[106] (2021)	△	✓	✓	Explores the concept and research of FL, specifically its application in confidential healthcare datasets.	The heterogeneity problem was not effectively addressed.
[107] (2022)	△	✓	✓	Covers the recent advancements of FL in smart healthcare. It introduces various designs including resource-aware, secure, privacy-aware, incentive-based, and personalised FL.	The topic of heterogeneity was not adequately addressed.
[9] (2020)	△	✓	✓	Provides the applications of mobile edge network optimisation, explains FL, analyses implementation challenges, evaluates existing solutions, reviews implementation difficulties, and considers potential future research paths.	Focuses on FL in mobile edge network optimisation, but does not explore it from a broader perspective.
[108] (2022)	✗	✓	✓	Focuses on image processing programs that ensure the safety and confidentiality of model training data.	The heterogeneity problem was not addressed.
[109] (2022)	△	✓	✓	Proposes a functional architecture for FL systems. The architecture includes components for parallelism, aggregation algorithms, data communication, and security. Additionally, the paper presents an overview of four widely used FL systems and summarises their limitations.	The issue of heterogeneity was not addressed effectively.
[110] (2022)	△	✓	✓	Propose novel applications of privacy-preserving FL.	Concentrated on addressing the mechanism for privacy preserving; it did not include a thorough taxonomy and discussion of the difficulties presented by heterogeneity.
[87] (2022)	✗	✓	✓	In-depth information about FL-based wireless communications applications is provided, emphasising key prerequisites, prospective uses, and difficulties in wireless networks.	The heterogeneity problem was not addressed.
[72] (2022)	△	✓	✓	In this work, they define and analyse non-IID data issues and offer a thorough investigation for resolving the issue, which poses significant statistical heterogeneity hurdles for FL.	Focuses on the challenges posed by statistical heterogeneity while ignoring other issues.
[4] (2022)	✗	✓	✓	Explores the research conducted to overcome communication constraints in a FL setting.	Does not effectively address the issue of heterogeneity.
[111] (2023)	△	✓	✓	Presents a complete survey of recent FL research, encompassing fundamentals, privacy and security procedures, communication overhead issues, heterogeneity issues, and practical applications.	The issues of heterogeneity were discussed from both a data and model perspective. However, a comprehensive classification and discussion of the challenges of heterogeneity were not provided.
[20] (2023)	✗	✓	✓	Thoroughly examines the challenges, solutions, and future directions of blockchain-empowered FL (BlockFed).	Does not effectively address the issue of heterogeneity.
[112] (2023)	✗	✓	✓	Explores the advantages of FL in medical applications, analysing security risks and attacks, and introducing standard privacy protection methods and discussed that when FL, combined with blockchain, edge computing, can enhance security and computational efficiency in healthcare applications.	Does not effectively address the issue of heterogeneity

## Data Availability

The data presented in this study are available on request from the corresponding author.
